# Benzenesulfonamides with different rigidity-conferring linkers as carbonic anhydrase inhibitors: an insight into the antiproliferative effect on glioblastoma, pancreatic, and breast cancer cells

**DOI:** 10.1080/14756366.2022.2091557

**Published:** 2022-06-29

**Authors:** Francesco Liguori, Simone Carradori, Roberto Ronca, Sara Rezzola, Serena Filiberti, Fabrizio Carta, Marta Turati, Claudiu T. Supuran

**Affiliations:** a Department of Pharmacy, “G. d’Annunzio” University of Chieti-Pescara, Chieti, Italy; b Neurofarba Department, University of Florence, Florence, Italy; c Department of Molecular and Translational Medicine, University of Brescia, Brescia, Italy

**Keywords:** Imidazolidine-2-one, ureido linker, carbonic anhydrase inhibitors, glioblastoma, pancreatic hypoxia, breast hypoxia, imidazolin-2-one

## Abstract

Among the chemotypes studied for selective inhibition of tumour-associated carbonic anhydrases (CAs), **SLC-0111**, a ureido-bearing benzenesulfonamide CA IX inhibitor, displayed promising antiproliferative effects in cancer cells *in vitro* and *in vivo*, being in Phase Ib/II clinical development. To explore the structural characteristics required for better discrimination of less conserved regions of the enzyme, we investigate the incorporation of the urea linker into an imidazolidin-2-one cycle, a modification already explored previously for obtaining CA inhibitors. This new library of compounds inhibited potently four different hCAs in the nanomolar range with a different isoform selectivity profile compared to the lead **SLC-0111**. Several representative CA IX inhibitors were tested for their efficacy to inhibit the proliferation of glioblastoma, pancreatic, and breast cancer cells expressing CA IX, in hypoxic conditions. Unlike previous literature data on **SLC-149**, a structurally related sulphonamide to compounds investigated here, our data reveal that these derivatives possess promising anti-proliferative effects, comparable to those of **SLC-0111**.

## Graphical Abstract

**Figure:**
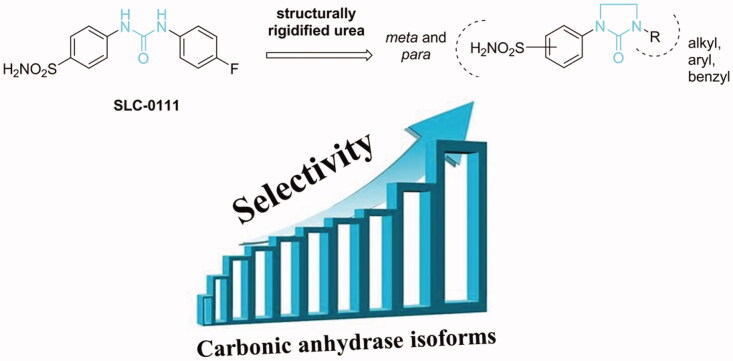

## 1. Introduction

Carbonic anhydrases (CAs, E.C. 4.2.1.1) are among the most studied metalloenzymes in Medicinal Chemistry. They are grouped into eight gene families and are present both in eukaryotes and prokaryotes^1^. In such organisms, they catalyse the carbon dioxide (CO_2_) interconversion into bicarbonate (HCO_3_^−^) and proton (H^+^) useful to physiologically/pathologically modulate pivotal cell processes, such as transport of CO_2_/HCO_3_^−^ between lungs and metabolising tissues, electrolyte secretion in tissues/organs using CO_2_ and pH homeostasis, bone remodelling, metabolic reactions (lipogenesis, gluconeogenesis, and ureagenesis), and tumorigenicity, at least in vertebrates^1^^,^^2^. The large plethora of therapeutic applications, e.g. epilepsy, glaucoma, idiopathic intracranial hypertension, and altitude sickness, with many inhibitors in the clinical use, allowed the wide exploration of CA modulators (inhibitors and activators) through the design of novel chemical scaffolds/chemotypes^3^^,^^4^. Accumulating evidence over the last two decades demonstrated the crucial role of the membrane-bound CA IX and XII (also called tumour-associated CAs) in the maintenance of a favourable intra-/extra-cellular pH for tumour cell survival and growth. Their hypoxia-inducible factors 1α (HIF-1α)-mediated overexpression as well as tissue/cell localisation pinpointed that these isozymes are responsible for cancer cell migration, invasion, and maintenance of stemness through the development of chemoresistance and in malignant progression^5–7^. Indeed, they can be viciously stimulated by and then support a tumour microenvironment characterised by hypoxia and extracellular acidosis hampering therapeutic response and altering cancer cell biology^8–10^. Moreover, in this acidic and hypoxic milieu tumour cells have been reported to gain increased genetic instability and mutagenesis rate^11^^,^^12^, and to become more resistant to radiation therapy and chemotherapy^13–16^. It has been amply demonstrated in several cell lines (melanoma, gastric, breast, oral, cervical, bladder, glioblastoma, pancreatic, hepatocellular, and colorectal cancer)^17–21^ and in xenograft tumours *in vivo*^22^^,^^23^ that selective CA IX and XII inhibitors can impair CA IX activity, triggering thus apoptosis and ferroptosis^23^.

**Figure 1.**
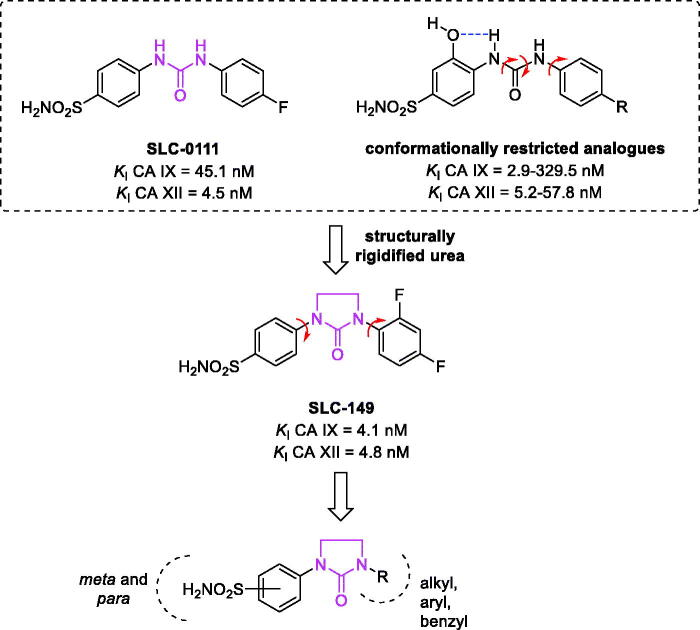
Previously described benzenesulfonamide ureas and rational approach to the design of the newly proposed structurally rigidified ureido compounds.

Among the several chemotypes studied for selective inhibition of tumour-associated CAs, the licenced **SLC-0111** (Figure 1), a ureido-bearing *p*-benzenesulfonamide CA IX inhibitor, is in Phase Ib/II clinical trial in combination with gemcitabine in pancreatic ductal adenocarcinoma patients with expression of CA IX (for both dose escalation and dose expansion) (https://clinicaltrials.gov/show/NCT03450018)^24^. The compound displayed promising antiproliferative effects in cancer cells transiently and chronically stimulated by extracellular acidosis, without exerting cytotoxicity in the cell population at standard pH conditions, and also enhanced ferroptosis due to the intracellular pH alkalinization reversal^23^. Moreover, it can be associated with conventional chemotherapy (dacarbazine, temozolomide, doxorubicin, 5-fluorouracil) or radiotherapy to significantly reduce tumour growth *in vivo*^25–28^.

As typical of several CAIs, **SLC-0111** has the benzenesulfonamide scaffold, with the sulfonamidate zinc-binding group (ZBG) being responsible for the potent nanomolar coordination of the zinc ion present in the active site. To overcome the lack of isoform-selectivity for first and second-generation sulphonamide CAIs, we often resort to a chemical strategy, known as the tail approach, providing better discrimination of less conserved regions of the enzyme and laid the groundwork for an extensive exploration of linkers between the ZBG and the tail. The insertion of a cyclic urea substituent on the phenyl ring of the benzenesulfonamide scaffold allows the entire molecule to better allocate within the enzymatic CA cavities proving a flexible interconnection with respect to the carboxyamido and sulphonamide groups^29–31^. Moreover, the urea functionality displays a high degree of rotational freedom, thus not blocking the molecule into a too much rigid system. Furthermore, attempts to partially restrict this structural feature were performed by designing derivatives bearing an OH group in proximity (*ortho*) to the benzenesulfonamide to form a stable intramolecular five-membered ring by H-bond with the ureido NH moiety^32^. The resulting conformationally restricted ureas (Figure 1) provided a preferential rotational isomer to interact within the CAs active sites displaying selectivity towards tumour-associated isoforms. However, when the urea was incorporated into the cyclic imidazolidin-2-one, leading to the structurally rigidified compound **SLC-149** (Figure 1), a reduction of isoform selectivity was observed compared to **SLC-0111** and some of its congeners^33^.

To better explore the chemical space within the ureido benzenesulfonamide scaffold and to study the feasibility of the synthetic scheme to a larger number of derivatives, we attempted to synthesise a library of compounds characterised by: (i) a primary benzenesulfonamide as ZBG; (ii) an imidazolidin-2-one linker in *para* or *meta* position with respect to the sulphonamide function; (iii) a tail characterised by chemical diversity in terms of electronic and steric effects (aliphatic chains, differently substituted aryl groups, benzyl moieties) (Figure 1). The insertion of the cyclic ureido moiety, already reported by Zhang^34^ and by Mboge et al.^33^, resulted in particular interest, both from the chemical and pharmacological viewpoints, as controversial results were claimed in some recent works^33^, according to which sulphonamides, such as **SLC-149** and congeners do not show antiproliferative activity and that the CA IX in the used cell lines does not possess a catalytic role in hypoxic tumours^33^.

## 2. Results and discussion

### 2.1. Rationale of the work: design and synthesis

**Scheme 1.**
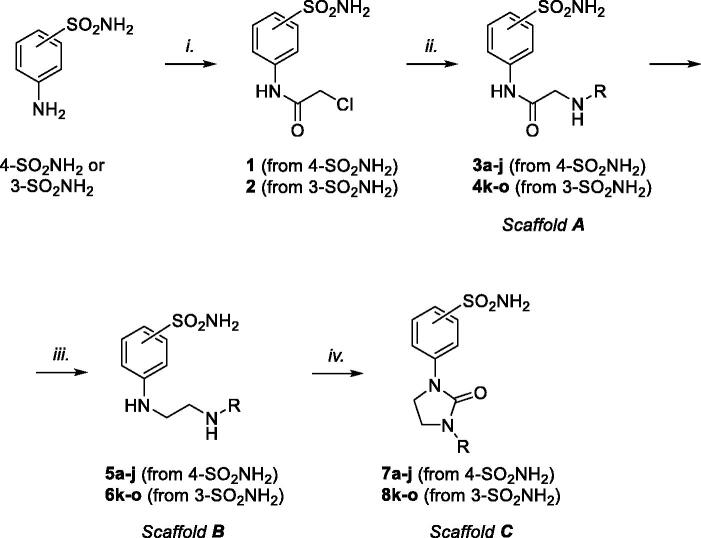
General synthetic approach to prepare compounds libraries endowed with scaffolds **A**, **B**, and **C**. For R substituents, please refer to Table 1. Reagents and conditions: (i) chloroacetyl chloride, dry acetone, N_2_, 0 °C, 0.5 h; (ii) for **3** and **4**: appropriately substituted aniline, KI, sealed tube, dry THF, N_2_, 110 °C, 24 h; for **3i**: 2-amino-6-methylpyridine, dry TEA, abs EtOH, N_2_, ref., 24 h; for **3j**: benzylamine, dry TEA, dry ACN, N_2_, 24 h; for **3a**, **3b**, and **4k**: *n*-butylamine or *i*-propylamine, KI, dry THF, N_2_, 24 h; (iii) for **5b**,**c**,**e**,**f**,**g**,**h** and **6l**: 1 M BH_3_·THF, dry THF, N_2_, r.t., 24 h; for **5a**,**d**,**i**,**j** and **6k**,**m**,**n**,**o**: LiAlH_4_, dry THF, N_2_, 0–70 °C, 24 h; *iv.* triphosgene, dry TEA, dry THF, N_2_, r.t., 2 h.

The preparation of ureido compounds (referred to scaffold **C** in Scheme 1) required the synthesis of amido (scaffold **A**) and amino (scaffold **B**) intermediates, as described in the following paragraphs. The different chemo-physical properties and rigidity of these scaffolds encouraged us to investigate their inhibitory activity profile against hCAs and, in particular, how the change in flexibility could affect the interaction with the target enzymes. Thus, all the final compounds and their corresponding intermediates were tested *in vitro* against two ubiquitous isoforms (hCA I and hCA II) and two membrane-anchored isoforms (hCA IX and hCA XII) to assess selectivity and inhibitory potency. In this work, we report the synthesis and characterisation of new sulphonamide ureido derivatives and report their antiproliferative activity *in vitro* on tumour cells representing glioblastoma, pancreatic, and breast cancer. The whole library of sulphonamides was obtained by a high-yielding multi-step synthetic approach, adapting previously reported pathways^34^^,^^35^, as reported in Scheme 1.

Both the *para-* and *meta-*series were obtained through the same synthetic strategy by using the commercially available 4- or 3-aminobenzenesulfonamide, respectively. Their anilino groups were reacted with chloroacetyl chloride in dry acetone, furnishing the chloroacetamides **1** and **2**, respectively. Then, a nucleophilic substitution with the appropriate aniline or amine yielded derivatives **3a–j** and **4k–o**, endowed with an amido scaffold **A**. Some changes in reaction conditions were required to furnish the whole set of amido compounds.

In brief, compounds **3a**,**b** and **4k**, endowed with aliphatic amine tails, were obtained in Finkelstein-like conditions by using potassium iodide in tetrahydrofuran (THF) at reflux, while, higher temperatures up to 110 °C were required for the anilino-bearing compounds. Otherwise, the same protocols resulted to be ineffective in preparing compounds **3i** and **3j**, bearing substituted pyridine and benzyl functions, respectively. Thus, we resorted to a traditional reaction with triethylamine (TEA) as a base. The amino derivatives **5a–j** and **6k–o** (scaffold **B**) were obtained by reducing the corresponding amides by using borane in the THF complex or lithium aluminium hydride. In the end, final compounds **7a–j** and **8k–o** (scaffold **C**) were afforded through a one-pot two-steps carbamoylation and intramolecular carbamoyl chloride-amino coupling *via* triphosgene and TEA as a base.

### 2.2. *In vitro* inhibition of human CAs and preliminary SARs consideration

The inhibition profiles for sulphonamides of scaffolds **A**, **B**, and **C** and the reference acetazolamide (**AAZ**) against the physiologically relevant hCAs I, II, IX, and XII isoforms were determined through the stopped-flow CO_2_ hydrase assay^36^ and are reported in Table 1.

**Table 1.** Inhibition data of all the synthesised sulphonamides and reference compound acetazolamide (**AAZ**) on hCA I, II, IX, and XII isoforms through the stopped-flow CO_2_ hydrase assay.

Cpd	Scaffold	Series	*R*	*K*_I_			
				hCA I [nM]	hCA II [nM]	hCA IX [nM]	hCA XII [nM]
**3a**	**A**	*p-*	*i*-propyl	92.0	26.6	32.8	140.0
**3b**	**A**	*p-*	*n*-butyl	160.5	8.4	355.3	171.3
**3c**	**A**	*p-*	phenyl	62.6	3.2	253.7	159.6
**3d**	**A**	*p-*	2-fluorophenyl	18.4	4.8	22.0	95.6
**3e**	**A**	*p-*	2-bromophenyl	29.8	3.0	341.8	161.0
**3f**	**A**	*p-*	4-tolyl	6.6	82.4	209.2	164.3
**3g**	**A**	*p-*	4-chlorophenyl	16.7	6.0	72.1	47.2
**3h**	**A**	*p-*	4-iodophenyl	>1000	909.3	68.0	174.7
**3i**	**A**	*p-*	6-methylpyridin-2-yl	15.3	4.7	38.3	162.3
**3j**	**A**	*p-*	benzyl	26.5	5.0	52.7	94.3
**4k**	**A**	*m-*	*n*-butyl	83.7	37.4	324.4	136.8
**4l**	**A**	*m-*	phenyl	443.3	35.5	302.2	152.3
**4m**	**A**	*m-*	2-bromophenyl	20.3	86.0	46.1	139.5
**4n**	**A**	*m-*	4-tolyl	86.4	8.2	66.7	158.0
**4o**	**A**	*m-*	4-chlorophenyl	268.7	32.4	72.1	47.2
**5a**	**B**	*p-*	*i*-propyl	896.0	337.7	25.7	85.3
**5b**	**B**	*p-*	*n*-butyl	538.1	82.2	64.8	119.1
**5c**	**B**	*p-*	phenyl	25.4	7.2	302.1	180.4
**5d**	**B**	*p-*	2-fluorophenyl	75.3	8.2	25.7	141.6
**5e**	**B**	*p-*	2-bromophenyl	939.2	908.0	380.5	160.5
**5f**	**B**	*p-*	4-tolyl	9.7	3.1	63.4	175.4
**5g**	**B**	*p-*	4-chlorophenyl	39.0	47.0	224.4	149.0
**5h**	**B**	*p-*	4-iodophenyl	786.6	645.8	204.7	153.2
**5i**	**B**	*p-*	6-methylpyridin-2-yl	87.0	31.4	47.4	170.1
**5j**	**B**	*p-*	benzyl	169.3	32.7	61.7	161.1
**6k**	**B**	*m-*	*n*-butyl	150.7	43.2	475.7	160.0
**6l**	**B**	*m-*	phenyl	7.0	4.1	52.4	166.7
**6m**	**B**	*m-*	2-bromophenyl	7.6	3.3	62.7	175.3
**6n**	**B**	*m-*	4-tolyl	>1000	900.7	411.8	166.7
**6o**	**B**	*m-*	4-chlorophenyl	345.7	15.7	57.1	168.0
**7a**	**C**	*p-*	*i*-propyl	8.5	3.2	67.6	42.5
**7b**	**C**	*p-*	*n*-butyl	603.5	423.0	483.0	82.0
**7c**	**C**	*p-*	phenyl	54.4	4.3	673.3	20.0
**7d**	**C**	*p-*	2-fluorophenyl	7.8	3.1	68.1	25.1
**7e**	**C**	*p-*	2-bromophenyl	6.2	3.4	203.1	8.7
**7f**	**C**	*p-*	4-tolyl	16.1	3.7	504.0	70.6
**7g**	**C**	*p-*	4-chlorophenyl	194.3	46.3	557.3	38.3
**7h**	**C**	*p-*	4-iodophenyl	92.7	13.0	477.3	54.0
**7i**	**C**	*p-*	6-methylpyridin-2-yl	54.6	7.2	300.0	51.4
**7j**	**C**	*p-*	benzyl	8.0	3.1	502.0	67.2
**8k**	**C**	*m-*	*n*-butyl	36.3	8.0	400.0	83.2
**8l**	**C**	*m-*	phenyl	60.6	31.1	607.6	75.4
**8m**	**C**	*m-*	2-bromophenyl	8.0	54.4	68.2	81.2
**8n**	**C**	*m-*	4-tolyl	461.6	500.0	559.2	93.4
**8o**	**C**	*m-*	4-chlorophenyl	30.3	84.1	468.0	>10000
**AAZ** (Ref. Compound)				250	12.1	25.7	5.7

*K*_I_ values are reported as means of three independent experiments by a stopped-flow technique. Errors are in the range of ±5–10% of the reported values. Acetazolamide (**AAZ**) was used as a reference control in these assays. Compounds are presented based on the molecular scaffold (**A**, amide; **B**, amine, and **C**, urea) and the (*meta*- or *para*-) position with respect to the benzenesulfonamide core.

Observing the reported data, several structure-activity relationship (SAR) considerations can be done based on the specific isoforms, the selectivity, and the chemical structures:

- Almost all the synthesised derivatives show a nanomolar inhibitory activity against hCA I, which was found higher than the reference compound **AAZ** in some cases. Among the amido compounds (scaffold **A**) with the sulphonamide function in *para*, the phenyl derivative **3c** shows a moderate-high activity that is partially increased with the introduction of chlorine (**3g**), bromine (**3e**), or iodine (**3d**) atoms in C4 or C2. Conversely, methyl (**3f**) and iodo (**3h**) substituents on the phenyl ring result to confer the best and the worst inhibitory activities, respectively, to the library. By comparing the same tails in the *meta-*series, an overall increase in the *K*_I_ values is observed, except for derivatives **4m** and **4k**. The switching from amide to amine (scaffold **B**) and the consequent decrease in structural rigidity seems to provoke significant changes in the SARs based on the molecular tails, with the exception of the iodophenyl derivative **5h** which is still among the less active derivatives. The *para*-series with alkyl substituents (**5b** and **5a**, bearing *n*-butyl and *i*-propyl groups, respectively) exerts a lower activity than **AAZ**, whereas differences can be noticed as regards the substituted phenyl derivatives. In fact, the insertion of a methyl group in C4 (**5f**) causes an increase in affinity with respect to the unsubstituted phenyl ring of **5c** *meta*-series, while the presence of a halogen atom overall worsens the inhibitory profile of such compounds. Although the 4-chloro (**5g**) and 2-fluoro (**5d**) substituents still maintain a good inhibitory activity on hCA I, higher than that of **AAZ**, the molecules functionalised with 4-iodo (**5h**) and 2-bromo (**5e**) phenyl groups inhibit hCA I with high-nanomolar concentrations, due to their bigger size. An opposite trend was recorded for the *meta*-series: sulphonamides **6l** and **6m**, bearing phenyl and 2-bromophenyl rings, respectively, are the best compounds in terms of activity, whereas the 4-tolyl derivative **6n** is totally inactive. In the end, among the cyclic ureas (scaffold **C**), 2-halophenyl (**7e** and **7d**) and benzyl (**7j**) along with *i*-propyl group (**7a**) are the best substituents for the *para-*series, while lower activities were noticed for the *meta*-series, except for the highly potent 2-bromophenyl derivative (**8m**).
- *K*_I_ values on hCA II reveal low nanomolar activities for the amido and urea derivatives (scaffolds **A** and **C**, respectively) of the *para*-series, following almost the same trend of data collected for the human isoform I. This could suggest that flexibility is not well-tolerated and causes a reduction in the enzyme inhibition for these derivative libraries. In general, apart from phenyl derivatives functionalised with the electron donor methyl group (**3f**) and the big nucleus of the iodine atom (**3h**), all the *para*-substituted compounds bearing aromatic rings possess a relevant activity on hCA II, with *K*_I_ values ranging from 3.0 to 6.0 nM. The aliphatic tailed derivative **3b** also shows a good inhibitory profile, while among *meta*-benzenesulfonamides, interesting results were shown only by compound **4n**. Observing data on the amino compounds (scaffold **B**), the phenyl ring seems to positively contribute to the inhibitory properties of both the *para-* and *meta-*series, while opposite results were obtained for the 4-tolyl (**5f** and **6n**) and 2-bromophenyl (**5e** and **6m**) derivatives. In fact, while **5f** belonging to the *para*-series and the *meta*-derivative **6m** are very potent inhibitors, the corresponding isomers **6n** and **5e** exert low activity on hCA II, suggesting the relevant role that the steric hindrance has in the affinity to this enzyme. The *para-*series with scaffold **C** emerged for very low *K*_I_ values, apart from the 4-chlorophenyl-bearing (**7h**) and the aliphatic tailed (**7b**) derivatives.
- Inhibition data against hCA IX are in the low-medium nanomolar range. In general, the linear precursors (scaffolds **A** and **B**) appear to have a better inhibitory profile towards this isoform than the corresponding ureido derivatives (scaffold **C**). In particular, the most active compounds belonging to scaffold **A** are: **3d**, **3i**, **3a**, and **4m**, while for scaffold **B** they are: **5i**, **5d**, and **5a**. It can therefore be noted that, both in the presence of the amide and diethylamine moieties, the best substituents are the isopropyl (**3a**, **5a**), the 2-halosubstituted phenyl ring with the fluorine (**3d**, **5d**) or bromine (**4m**) atoms or the presence of 6-methylpyridin-2-yl group (**3i**, **5i**). The same trend is noticed for ureido compounds **7a**, **7d**, and **8m**.
- Regarding hCA XII enzymatic activity, the compounds were very potent nanomolar inhibitors *in vitro* (except compound **8o**), without any marked difference between the *meta* and the *para* series. Scaffolds **A** and **B** are equipotently providing several promising compounds for further development. The most important result is the enhancement of the hCA XII inhibitory activity after cyclisation of the ureidic linker. Indeed, all compounds (except **8o**) displayed *K*_I_ values inferior to 93.4 nM achieving 8.7 nM for compound **7e**. The sulphonamide group was preferred when in the *para* position.
- Collectively, more satisfactory data are obtained from the molecules with scaffold **C**, therefore with a cyclic ureidic portion, rather than from the ureidic and ethylamine portions (scaffolds **A** and **B**). In fact, among amido derivatives (scaffold **A**), the only ones endowed with *K*_I_ values below 100 nM are those with the 4-chlorophenyl portion both in the *para* (**3g**) and *meta* (**4o**) position, the 2-fluorophenyl ring (**3e**), and the benzyl moiety (**3j**). Scaffold **B**-bearing most active molecule is **5a**, with an isopropyl portion, while among the ureido compounds (scaffold **C**), all the derivatives showed a *K*_I_ lower than 100 nM, with the exception of the compound **8o**. In detail, compound **7e**, characterised by a 2-bromophenyl portion in the *para* position with respect to the sulphonamide function, is the one that exerts a similar activity to that of the reference standard **AAZ**.

### 2.3. Evaluation of benzenesulfonamide CAIs effects on glioblastoma cancer cells

**Figure 2.**
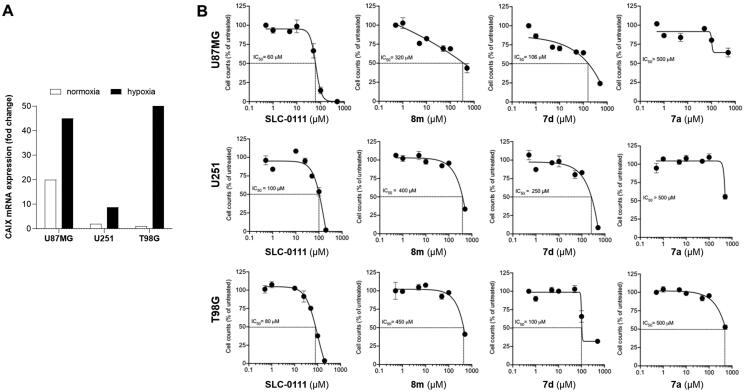
(A) Gene expression levels (mRNA) of CA IX in glioblastoma cancer cells under normoxic or hypoxic conditions and (B) cell proliferation of these cells treated with compounds **8m**, **7d**, **7a**, or **SLC-0111** (cell count is referred to the untreated/control considered as 100%).

To evaluate the effect of CAIs in human cancer cells, the expression of CA IX was first evaluated by quantitative PCR in three different glioblastoma cell lines cultured in normoxic and hypoxic (1% O_2_, 5% CO_2_, and 94% N_2_) conditions for 24 h. As shown in Figure 2, mRNA levels of CA IX are significantly increased after exposure to hypoxia for 24 h.

Then, we evaluated the efficacy of the three ureido CA IX inhibitors, as representatives of the whole derivatives library with scaffold **C**, **7a**, **7d**, and **8m** to inhibit the proliferation of these glioblastoma cells after 72 h of treatment in hypoxia. To compare the results of the experiment, we also tested **SLC-0111** as a reference compound. As shown in Figure 2, the clinical grade compound **SLC-0111** was effective in reducing the proliferation of U87MG, U251, and T98G cell lines, with an IC_50_ ranging from 60 to 100 µM. Interestingly, even if less effective than **SLC-0111**, all the three compounds exhibited anti-proliferative activity, compound **7d** resulting the most effective in comparison with compounds **8m** and **7a**, with an IC_50_ ranging from 100 to 250 µM.

These data suggest that glioblastoma cells are sensitive to the inhibition of CA IX and that compound **7d** has a promising anti-proliferative effect in this tumour context.

### 2.4. Evaluation of CAIs effects on pancreatic cancer cells

**Figure 3.**
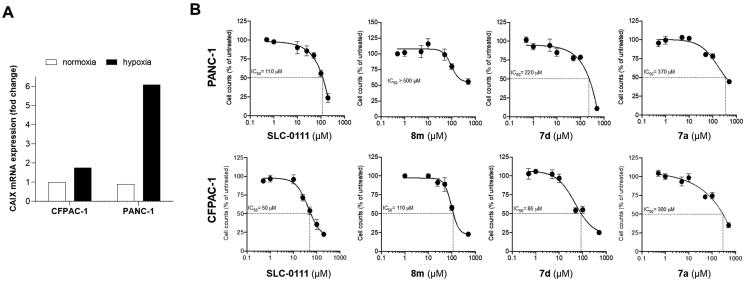
(A) Gene expression levels (mRNA) of *CA IX* in pancreatic cancer cells under normoxic or hypoxic conditions and (B) cell proliferation of these cells treated with compounds **8m**, **7d**, **7a**, or **SLC-0111** (cell count is referred to the untreated/control considered as 100%).

The expression levels of *CA IX* were assessed in two cell lines of pancreatic cancer (CF-PAC-1 and PANC-1 cells) under hypoxic conditions, revealing a significant upregulation of the gene expression (Figure 3).

As shown in Figure 3 all the investigated CAIs (**7a**, **7d**, and **8m**) were efficacious in reducing pancreatic cancer cell proliferation, compound **7d** resulting the most effective with an IC_50_ of 85 µM on CFPAC-1 cells and 220 µM on PANC-1 cells. Indeed, the reference compound **SLC-0111** resulted to be the most effective with an IC_50_ of 50 µM on CFPAC-1 cells and 110 µM on PANC-1 cells. These data confirm the efficacy of **SLC-0111** on pancreatic cancer cells and the fact that compound **7d** has a higher effect compared to the other derivatives, also on this tumour type.

### 2.5. Evaluation of CAIs effects on breast cancer cells

**Figure 4.**
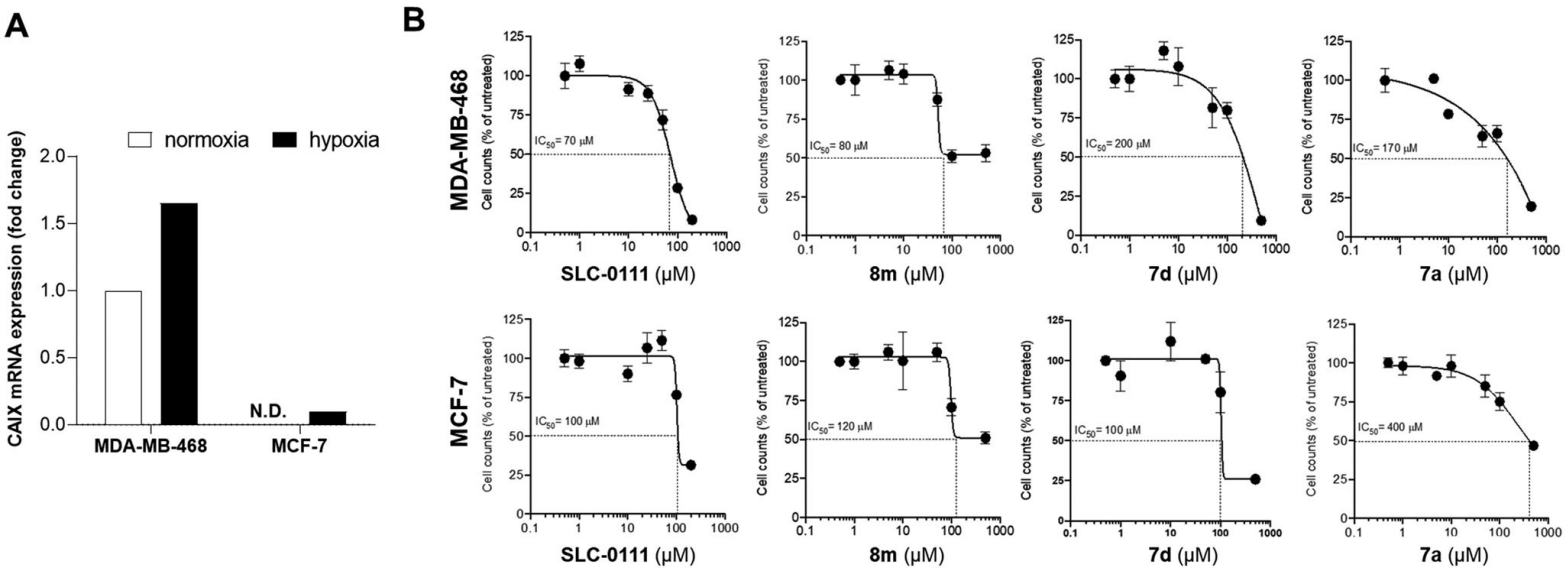
(A) Gene expression levels (mRNA) of *CA IX* in breast cancer cells under normoxic or hypoxic conditions and (B) cell proliferation of these cells treated with compounds **8m**, **7d**, **7a**, or **SLC-0111** (cell count is referred to the untreated/control considered as 100%).

In line with the previous experiments, we verified the expression of *CA IX* in two cell lines of breast cancer (MDA-MB-468 and MCF-7 cells) under hypoxic conditions and confirmed that in MDA-MB-468 cells there is an increase in CA IX expression, while in MCF-7 cells hypoxic conditions trigger the expression of CA IX that was undetectable in normoxia (Figure 4).

*In vitro* evaluation of the CAIs revealed that also in these cell lines, **SLC-0111** reduced cell proliferation with an IC_50_ of 70 µM on MDA-MB-468 and 100 µM on MCF-7 cells. Compound **7d** resulted in efficacious with an IC_50_ of 200 µM on MDA-MB-468 and of 100 µM on MCF-7 cells and, in this case, it was less effective than compound **8m**, even if the latter does not reach total inhibition of cell growth at the higher concentration tested. In general, compound **7a** resulted to be less effective on the breast cancer cells tested.

## 3. Conclusion

Keeping in mind that the ureidic linker was successfully applied to the synthesis of potent CA IX and XII inhibitors, we followed a medicinal chemistry approach aimed at investigating how the cyclisation of this moiety into a rigidified imidazolidin-2-one group could impact CA inhibitory activity, isoform selectivity *in vitro*, and pharmacokinetic characteristics, considering the existing controversy in the field^33^^,^^37^. The introduction of the rigid imidazolidin-2-one linker was associated with a loss of selectivity for inhibiting CA IX and XII over CA II, but these compounds did possess interesting inhibition profiles. Thus, the three most representative compounds were assayed against several cancer cell lines under hypoxic conditions in comparison with **SLC-0111**. Enzyme inhibition results confirmed that chemical manipulation of the linker and the tail could modulate the CA activity, despite the presence of a pan-CA inhibiting zinc binding group (sulphonamide). Cell-based data confirmed the relevant antiproliferative properties of this class of CAIs, the importance of the tumour-associated CAs for tumour growth and development, and their inhibition as a fruitful strategy to hamper these diseases, reinforcing previous data^37^^,^^38^ on the significant antitumor effects of this class of CA inhibitors.

## 4. Materials and methods

### 4.1. Chemistry

#### 4.1.1. General chemistry

All anhydrous solvents and reagents were purchased from Alfa Aesar, TCI, and Merck. The synthetic reactions involving air- or moisture-sensitive chemicals were carried out under a nitrogen atmosphere using flame-dried glassware and syringe techniques to transfer the solutions. Analytical thin-layer chromatography (TLC) was performed on Merck silica gel F-254 plates. TLCs were visualised under UV light and stained with ninhydrin stain. Chromatographic separations were performed on columns packed with Merck Silica gel 60 (230–400 mesh ASTM, for flash technique) as the stationary phase. Melting points (m.p.) were measured in open capillary tubes with a Gallenkamp MPD350.BM3.5 apparatus and are uncorrected.

Nuclear magnetic resonance (^1^H and ^13^C NMR) spectra were recorded using a Bruker Advance III 400 MHz spectrometer at 400 and 100 MHz, respectively using DMSO-*d*_6_ as solvent. The chemical shifts are reported in parts per million (ppm) and internally referenced to DMSO-*d*_6_ signal at δ 2.50 and 39.0 ppm for ^1^H and ^13 ^C NMR spectra, respectively. Data are shown as follows: chemical shift, multiplicity (splitting patterns are designated as s, singlet; d, doublet; t, triplet; q, quartette; m, multiplet; brs, broad singlet; dd, doublet of doublets; ap, apparent), integration, and coupling constants (*J*) in Hertz (Hz). The correct assignment of exchangeable protons (i.e. OH and NH) was carried out using the addition of D_2_O.

The high-resolution mass spectrometry (HRMS) analysis was performed using a Thermo Finnigan LTQ Orbitrap mass spectrometer coupled with an electrospray ionisation source (ESI). The HRMS analysis was performed by introducing the analyte working solution *via* syringe pump at 10 μL/min. Analyses were carried out in positive ion scanning mode and it was used a proper dwell time acquisition to achieve 60.000 units of resolution at full width at half maximum (FWHM). The elemental composition of compounds was calculated based on their measured accurate masses, accepting only results with an attribution error <5 ppm and a not integer RDB (double bond/ring equivalents) value (data not shown). Stock solutions of analytes were prepared using acetone (1.0 mg/mL) and stored at 4 °C. Then, working solutions of each analyte were prepared by dilution of the stock solutions using mQ H_2_O/ACN 1/1 (*v/v*) up to a concentration of 1.0 μg/mL.

The behaviour of all the tested compounds as pan-assay interference compounds (PAINS) was examined through prediction by the SwissADME tool^39^, revealing the absence of PAINS fragments (0 alert), and the online FAFDrugs4 (Free ADME-Tox Filtering Tool) program^40^ (for non-fluorine-bearing compounds). Through its tool Bank-Formatter, the compound library was prepared and then screened with the three different available filters A, B, and C^41^. All the analysed compounds resulted as “accepted” by the software (FAFDrugs4, https://fafdrugs4.rpbs.univ-paris-diderot.fr/).

### 4.2. General procedure for compounds 1 and 2

4-Aminobenzensulfonamide **1** or 3-aminobenzensulfonamide **2** (2.00 g, 11.61 mmol, 1 eq.) and potassium carbonate (2.08 g, 15.09 mmol, 1.3 eq.) were suspended in dry acetone (20 mL) under nitrogen atmosphere and the suspension was cooled to 0 °C in an ice bath. Chloroacetyl chloride (924 μL, 11.61 mmol, 1 eq.) was added dropwise under a nitrogen atmosphere. The reaction was stirred for 30 min at room temperature, quenched with water, filtered and the filtrate was evaporated under vacuum. The product was obtained with no further purification.

#### 4.2.1. 2-Chloro-N-(4-sulfamoylphenyl)acetamide (1)

White solid. Yield = 69%, silica gel TLC R_f_ 0.50 (EtOAc/Hex 70% *v/v*); ^1^H NMR (400 MHz, DMSO-*d*_6_) *δ* (ppm): 7.76 (m, 4H), 7.26 (s, 2H), 4.29 (s, 2H).

#### 4.2.2. 2-Chloro-N-(3-sulfamoylphenyl)acetamide (2)

White solid. Yield = 69%, silica gel TLC R_f_ 0.50 (EtOAc/Hex 70% *v/v*); ^1^H NMR (400 MHz, DMSO-*d*_6_) *δ* (ppm): 10.67 (s, 1H), 8.19 (s, 1H), 7.78 (dd, 1H, *J =* 6.42 Hz), 7.57 (d, 2H, *J =* 6.42 Hz), 7.43 (s, 2H), 4.33 (s, 2H).

### 4.3. General procedure for compounds 3a–h and 4k–o

To a stirring solution of **1** or **2** (1.00 g, 4.02 mmol, 1 eq.) in dry THF in a tube, KI (100.0 mg, 0.60 mmol, 0.15 eq.) for **3a–h** or (200.0 mg, 1.20 mmol, 0.30 eq.) for **4k–o** and the appropriate amine or aniline (24.12 mmol, 6 eq.) were added at room temperature under nitrogen atmosphere. Then, the reaction mixture was heated at 110 °C for 24 h. After reaction completion, the reaction was quenched with deionised water and extracted with EtOAc three times. The combined organic layers were washed with brine, dried over anhydrous Na_2_SO_4_, filtered, and evaporated under vacuum. The obtained crudes were washed with diethyl ether and purified by silica gel flash column chromatography.

#### 4.3.1. 2-(Isopropylamino)-N-(4-sulfamoylphenyl)acetamide (3a)

Red solid. Yield = 39%, silica gel TLC R_f_ 0.10 (MeOH/DCM 15% *v/v*); ^1^H NMR (400 MHz, DMSO-*d*_6_) *δ* (ppm): 10.19 (bs, 1H), 7.82 (m, 4H), 7.28 (s, 2H), 3.37 (s, 2H), 2.79 (m, 1H), 1.05 (d, 6H, *J =* 6.24 Hz); ^13 ^C NMR (100 MHz, DMSO-*d*_6_) *δ* (ppm): 172.3, 142.5, 139.8, 127.8, 119.7, 51.6, 49.3, 23.6; HRMS *m*/*z*: 272.1 [M + H]^+^.

#### 4.3.2. 2-(Butylamino)-N-(4-sulfamoylphenyl)acetamide (3b)

Yellow solid. M.p. 167–169 °C. Yield = 96%, silica gel TLC R_f_ 0.15 (MeOH/DCM 15% *v/v*); ^1^H NMR (400 MHz, DMSO-*d*_6_) *δ* (ppm): 10.09 (bs, 1H), 7.81 (dd, 4H, *J =* 8.94 Hz, *J =* 7.13 Hz), 7.27 (s, 2H), 3.37 (s, 2H), 2.57 (d, 2H, *J =* 6.89 Hz), 1.44 (m, 2H), 1.35 (m, 2H), 0.91 (t, 3H, *J =* 7.31 Hz); ^13 ^C NMR (100 MHz, DMSO-*d*_6_) *δ* (ppm): 171.6, 142.0, 138.8, 127.1, 119.1, 53.4, 49.3, 32.1, 20.3, 14.3; HRMS *m*/*z*: 286.1 [M + H]^+^.

#### 4.3.3. 2-(Phenylamino)-N-(4-sulfamoylphenyl)acetamide (3c)

White solid. M.p. 123–125 °C. Yield =59%, silica gel TLC R_f_ 0.2 (MeOH/DCM 5% *v/v*); ^1^H NMR (400 MHz, DMSO-*d*_6_) *δ* (ppm): 10.35 (s, 1H), 7.80 (dd, 4H, *J =* 9.34 Hz, *J =* 15.52 Hz), 7.28 (s, 2H), 7.13 (t, 2H, *J =* 7.44 Hz), 6.63 (d, 3H, *J =* 8.59 Hz), 6.59 (d, 1H, *J =* 5.02 Hz), 6.03 (t, 1H, *J =* 6.11 Hz), 3.93 (d, 2H, *J =* 6.16 Hz); ^13 ^C NMR (100 MHz, DMSO-*d*_6_) *δ* (ppm): 171.1, 149.3, 142.8, 139.5, 129.9, 127.8, 119.9, 117.6, 113.4, 48.5; HRMS *m*/*z*: 306.1 [M + H]^+^.

#### 4.3.4. 2-((2-Fluorophenyl)amino)-N-(4-sulfamoylphenyl)acetamide (3d)

Pink solid. M.p. 191–193 °C. Yield = 38%, silica gel TLC R_f_ 0.50 (EtOAc/Hex 70% *v/v*); ^1^H NMR (400 MHz, DMSO-*d*_6_) *δ* (ppm): 10.40 (s, 1H), 7.80 (s, 4H), 7.27 (s, 2H), 7.08 (t, 1H, *J =* 10.13 Hz), 7.00 (t, 1H, *J =* 7.67 Hz), 6.66 (t, 2H, *J =* 9.53 Hz), 5.79 (d, 1H, *J =* 6.36 Hz), 4.01 (d, 2H, *J =* 5.77 Hz); HRMS *m*/*z*: 324.1 [M + H]^+^.

#### 4.3.5. 2-((2-Bromophenyl)amino)-N-(4-sulfamoylphenyl)acetamide (3e)

Black solid. Yield = 49%, silica gel TLC R_f_ 0.27 (MeOH/DCM 3% *v/v*); ^1^H NMR (400 MHz, DMSO-*d*_6_) *δ* (ppm): 10.51 (s, 1H), 7.80 (s, 4H), 7.48 (dd, 1H, *J =* 8.14 Hz, *J =* 8.29 Hz), 7.29 (s, 2H), 7.23 (t, 1H, *J =* 7.35 Hz), 6.62 (d, 2H, *J =* 5.92 Hz), 5.62 (t, 1H, *J =* 6.39 Hz), 4.08 (d, 2H, *J =* 5.59 Hz); ^13 ^C NMR (100 MHz, DMSO-*d*_6_) *δ* (ppm): 169.4, 145.1, 142.1, 138.9, 132.7, 129.2, 127.2, 119.2, 118.3, 112.1, 109.2, 47.3; HRMS *m*/*z*: 384.0 [M + H]^+^.

#### 4.3.6. N-(4-sulfamoylphenyl)-2-(p-tolylamino)acetamide (3f)

Brown solid. Yield = 51%, silica gel TLC R_f_ 0.20 (MeOH/DCM 5% *v/v*); ^1^H NMR (400 MHz, DMSO-*d*_6_) *δ* (ppm): 10.30 (s, 1H), 7.80 (dd, 4H, *J =* 11.53 Hz, *J =* 15.01 Hz), 7.27 (s, 2H), 6.94 (d, 2H, *J =* 8.21 Hz), 6.55 (d, 2H, *J =* 8.21 Hz), 5.84 (t, 1H, *J =* 5.92 Hz), 3.89 (d, 2H, *J =* 5.99 Hz), 2.18 (s, 3H); HRMS *m*/*z*: 320.1 [M + H]^+^ in agreement with^35^.

#### 4.3.7. 2-((4-Chlorophenyl)amino)-N-(4-sulfamoylphenyl)acetamide (3g)

Purple solid. M.p. 210–212 °C. Yield = 84%, silica gel TLC R_f_ 0.2 (MeOH/DCM 5% *v/v*); ^1^H NMR (400 MHz, DMSO-*d*_6_) *δ* (ppm): 10.37 (s, 1H), 7.80 (s, 4H), 7.28 (s, 2H), 7.15 (d, 2H, *J =* 8.30 Hz), 6.64 (d, 2H, *J =* 8.23 Hz), 6.26 (bs, 1H), 3.95 (s, 2H); ^13 ^C NMR (100 MHz, DMSO-*d*_6_) *δ* (ppm): 170.1, 147.7, 142.2, 138.9, 129.0, 127.2, 120.2, 119.3, 114.2, 47.6, 31.1; HRMS *m*/*z*: 340.0 [M + H]^+^ in agreement with^35^.

#### 4.3.8. 2-((4-Iodophenyl)amino)-N-(4-sulfamoylphenyl)acetamide (3h)

White solid. Yield = 58%, silica gel TLC R_f_ 0.15 (MeOH/DCM 5% *v/v*); ^1^H NMR (400 MHz, DMSO-*d*_6_) *δ* (ppm): 10.30 (s, 1H), 7.80 (dd, 4H, *J =* 11.53 Hz, *J =* 15.01 Hz), 7.27 (s, 2H), 6.94 (d, 2H, *J =* 8.21 Hz), 6.55 (d, 2H, *J =* 8.21 Hz), 5.84 (t, 1H, *J =* 5.92 Hz), 3.89 (d, 2H, *J =* 5.99 Hz); ^13 ^C NMR (100 MHz, DMSO-*d*_6_) *δ* (ppm): 170.8, 149.5, 142.9, 140.0, 129.9, 127.7, 119.6, 118.2, 113.4, 49.2; HRMS *m*/*z*: 431.9 [M + H]^+^.

#### 4.3.9. 2-(Butylamino)-N-(3-sulfamoylphenyl)acetamide (4k)

Yellow solid. Yield = 60%, silica gel TLC R_f_ 0.10 (MeOH/DCM 15% *v/v*); ^1^H NMR (400 MHz, DMSO-*d*_6_) *δ* (ppm): 10.09 (bs, 1H), 8.26 (s, 1H), 7.80 (d, 1H, *J =* 5.00 Hz), 7.54 (d, 2H, *J =* 5.90 Hz), 7.39 (bs, 2H), 2.12 (t, 1H, *J =* 7.50 Hz), 1.45 (m, 3H), 1.36 (m, 3H), 0.91 (t, 5H, *J =* 7.01 Hz); ^13 ^C NMR (100 MHz, DMSO-*d*_6_) *δ* (ppm): 171.9, 145.7, 140.1, 130.5, 123.1, 121.4, 117.2, 53.9, 49.8, 32.7, 20.9, 14.9; HRMS *m*/*z*: 286.1 [M + H]^+^.

#### 4.3.10. 2-(Phenylamino)-N-(3-sulfamoylphenyl)acetamide (4l)

Brown solid. M.p. 181–183 °C. Yield = 35%, silica gel TLC R_f_ 0.20 (MeOH/DCM 5% *v/v*); ^1^H NMR (400 MHz, DMSO-*d*_6_) *δ* (ppm): 10.32 (s, 1H), 8.22 (s, 1H), 7.82 (m, 1H), 7.53 (apt, 2H, *J =* 7.67 Hz), 7.38 (s, 2H), 7.13 (t, 2H, *J =* 7.44 Hz), 6.62 (apt, 3H, *J =* 8.65 Hz), 6.05 (t, 1H, *J =* 6.10 Hz), 3.92 (d, 2H, *J =* 6.23 Hz); ^13 ^C NMR (100 MHz, DMSO-*d*_6_) *δ* (ppm): 170.9, 149.3, 145.7, 140.2, 130.5, 130.0, 123.2, 121.5, 117.6, 117.4, 113.4, 48.4; HRMS *m*/*z*: 306.1 [M + H]^+^.

#### 4.3.11. 2-((2-Bromophenyl)amino)-N-(3-sulfamoylphenyl)acetamide (4m)

Purple solid. Yield = 49%, silica gel TLC R_f_ 0.33 (MeOH/DCM 5% *v/v*); ^1^H NMR (400 MHz, DMSO-*d*_6_) *δ* (ppm): 10.49 (s, 1H), 8.22 (s, 1H), 7.78 (s, 1H), 7.54 (d, 2H, *J =* 5.24 Hz), 7.48 (d, 1H, *J =* 8.14 Hz), 7.42 (s, 1H), 7.39 (s, 2H), 7.22 (t, 1H, *J =* 7.65 Hz), 6.61 (d, 1H, *J =* 7.47 Hz), 5.64 (t, 1H, *J =* 5.43 Hz), 4.06 (d, 2H, *J =* 5.53 Hz); ^13 ^C NMR (100 MHz, DMSO-*d*_6_) *δ* (ppm): 168.5, 146.8, 139.9, 138.8, 132.4, 128.5, 127.0, 124.8, 119.8, 115.7, 114.5, 54.5; HRMS *m*/*z*: 384.0 [M + H]^+^.

#### 4.3.12. N-(3-sulfamoylphenyl)-2-(p-tolylamino)acetamide (4n)

Yellow solid. Yield = 49%, silica gel TLC R_f_ 0.38 (MeOH/DCM 5% *v/v*); ^1^H NMR (400 MHz, DMSO-*d*_6_) *δ* (ppm): 10.27 (s, 1H), 8.21 (s, 1H), 7.81 (s, 1H), 7.45 (d, 4H, *J =* 10.13 Hz), 6.95 (d, 2H, *J =* 7.18 Hz), 6.55 (d, 2H, *J =* 8.28 Hz), 5.84 (d, 1H, *J =* 6.53 Hz), 3.88 (d, 2H, *J =* 6.67 Hz), 2.17 (d, 3H, *J =* 7.41 Hz); ^13 ^C NMR (100 MHz, DMSO-*d*_6_) *δ* (ppm): 171.1, 147.0, 145.7, 140.2, 130.5, 130.4, 126.0, 123.2, 121.4, 117.3, 113.5, 48.8, 21.1; HRMS *m*/*z*: 320.1 [M + H]^+^.

#### 4.3.13. 2-((4-Chlorophenyl)amino)-N-(3-sulfamoylphenyl)acetamide (4o)

Purple solid. M.p. 148–150 °C. Yield = 57%, silica gel TLC R_f_ 0.40 (MeOH/DCM 5% *v/v*); ^1^H NMR (400 MHz, DMSO-*d*_6_) *δ* (ppm): 10.34 (s, 1H), 8.21 (s, 1H), 7.80 (s, 1H), 7.54 (s, 2H), 7.38 (s, 2H), 7.15 (d, 2H, *J =* 8.48 Hz), 6.63 (d, 2H, *J =* 8.44 Hz), 6.27 (bs, 1H), 3.92 (d, 2H, *J =* 5.71 Hz); ^13 ^C NMR (100 MHz, DMSO-*d*_6_) *δ* (ppm): 170.5, 148.3, 145.7, 140.2, 130.5, 129.7, 123.2, 121.5, 120.9, 117.4, 114.8, 48.3; HRMS *m*/*z*: 340.0 [M + H]^+^.

### 4.4. Synthesis of 2-((6-methylpyridin-2-yl)amino)-N-(4-sulfamoylphenyl)acetamide (3i)

To a stirring solution of **2.1a** (1 g, 4.02 mmol, 1 eq.) in abs EtOH (25 mL), 6-methylpyridin-2-amine (434.51 mg, 4.018 mmol, 1 eq.) and dry TEA (0.56 mL, 4.02 mmol, 1 eq.) were added. Then, the reaction mixture was stirred at 78 °C for 16 h. After completion, 1 M HCl was added till neutralisation and the mixture was extracted with EtOAc three times. The combined organic layers were washed with brine, dried over anhydrous Na_2_SO_4_, filtered, and evaporated under a vacuum. The product was obtained with no further purification. Red solid. Yield = 46%, silica gel TLC R_f_ 0.65 (EtOAc/Hex 70% *v/v*); ^1^H NMR (400 MHz, DMSO-*d*_6_) *δ* (ppm): 10.68 (s, 1H), 7.81 (m, 4H), 7.33 (s, 2H), 7.27 (t, 1H, *J =* 7.56 Hz), 6.36 (d, 1H, *J =* 7.16 Hz), 6.26 (d, 1H, *J =* 8.30 Hz), 4.34 (s, 2H), 2.24 (s, 3H); ^13 ^C NMR (100 MHz, DMSO-*d*_6_) *δ* (ppm): 157.4, 151.0, 142.5, 128.5, 127.7, 119.7, 112.3, 109.1, 106.8, 46.2, 21.7; HRMS *m*/*z*: 321.1 [M + H]^+^.

### 4.5. Synthesis of 2-(benzylamino)-N-(4-sulfamoylphenyl)acetamide (3j)

**1** (1.00 g, 4.02 mmol, 1 eq.) was dissolved in dry ACN (20 ml) and then benzylamine (0.48 mL, 4.42 mmol, 1.1 eq.) and dry TEA (0.73 mL, 5.22 mmol, 1.3 eq.) were added under nitrogen atmosphere. The reaction was stirred at 82 °C for 16 h. After cooling, the reaction was quenched with water and filtered. The obtained solid was washed several times with Et_2_O. White solid. M.p. 195–1978 °C. Yield = 39%, silica gel TLC R_f_ 0.45 (MeOH/DCM 5% *v/v*); ^1^H NMR (400 MHz, DMSO-*d*_6_) *δ* (ppm): 10.20 (s, 1H), 7.81 (m, 4H), 7.37 (m, 4H), 7.28 (s, 2H), 3.78 (s, 2H), 3.35 (s, 2H); ^13 ^C NMR (100 MHz, DMSO-*d*_6_) *δ* (ppm): 171.3, 142.1, 140.7, 138.8, 128.7, 128.5, 127.2, 127.1, 119.2, 53.0, 52.5; HRMS *m*/*z*: 329.1 [M + H]^+^.

### 4.6. General procedure for compounds 5b,c,e,f,g,h, and 6l

To a stirring solution of the suitable amide derivative (**3b**,**c**,**e**,**f**,**g**,**h**, or **4l**) (150.0 mg, 0.47 mmol, 1 eq.) in dry THF (20 mL), 1 M BH_3·_THF complex (3.25 mL, 3.09 mmol, 6.5 eq.) was added at room temperature under nitrogen atmosphere. Then, the mixture was stirred at 110 °C for 24 h. After cooling, the reaction mixture was quenched with deionised water, and extracted with EtOAc three times. The combined organic layers were washed with brine, dried over anhydrous Na_2_SO_4_, filtered, and evaporated under vacuum. The reaction crudes were purified by silica gel flash column chromatography.

#### 4.6.1. 4-((2-(Butylamino)ethyl)amino)benzenesulfonamide (5b)

Yellow solid. M.p. 165–167 °C. Yield = 43%, silica gel TLC R_f_ 0.16 (MeOH/DCM 15% *v/v*); ^1^H NMR (400 MHz, DMSO-*d*_6_) *δ* (ppm): 7.81 (m, 4H), 7.28 (s, 2H), 3.51 (s, 4H), 1.81 (s, 1H), 1.45 (m, 2H), 1.37 (m, 2H), 1.27 (m, 2H), 0.93 (t, 3H, *J =* 6.04 Hz); ^13 ^C NMR (100 MHz, DMSO-*d*_6_) *δ* (ppm): 152.6, 130.9, 128.4, 111.8, 49.9, 49.2, 43.6, 32.9, 21.1, 15.0; HRMS *m*/*z*: 272.1 [M + H]^+^.

#### 4.6.2. 4-((2-(Phenylamino)ethyl)amino)benzenesulfonamide (5c)

White solid. Yield = 33%. silica gel TLC R_f_ 0.50 (EtOAc/Hex 70% *v/v*); ^1^H NMR (400 MHz, DMSO-*d*_6_) *δ* (ppm): 7.55 (d, 2H, *J =* 8.42 Hz), 7.11 (t, 2H, *J =* 7.64 Hz), 6.40 (s, 2H), 6.68 (s, 2H, *J =* 8.61 Hz), 6.62 (d, 2H, *J =* 7.91 Hz), 6.57 (t, 2H, *J =* 7.55 Hz), 6.46 (apt, 1H, *J =* 7.13 Hz); ^13 ^C NMR (100 MHz, DMSO-*d*_6_) *δ* (ppm): 152.4, 149.7, 131.2, 130.0, 128.5, 116.9, 113.2, 111.9, 42.9, 42.8; HRMS *m*/*z*: 292.1 [M + H]^+^.

#### 4.6.3. 4-((2-((2-Bromophenyl)amino)ethyl)amino)benzenesulfonamide (5e)

Brown solid. Yield = 69%, silica gel TLC R_f_ 0.24 (MeOH/DCM 3% *v/v*); ^1^H NMR (400 MHz, DMSO-*d*_6_) *δ* (ppm): 7.55 (d, 2H, *J =* 8.77 Hz), 7.44 (d, 1H, *J =* 7.82 Hz), 7.22 (t, 1H, *J =* 8.06 Hz), 6.96 (s, 2H), 6.78 (d, 1H, *J =* 7.82 Hz), 6.70 (d, 2H, *J =* 8.77 Hz), 6.56 (t, 2H, *J =* 6.54 Hz), 5.30 (apt, 1H, *J =* 5.45 Hz), 4.36 (s, 4H); ^13 ^C NMR (100 MHz, DMSO-*d*_6_) *δ* (ppm): 144.3, 143.1, 138.9, 132.7, 130.4, 129.5, 119.2, 117.3, 111.1, 109.2, 47.3, 42.7; HRMS *m*/*z*: 370.0 [M + H]^+^.

#### 4.6.4. 4-((2-(p-Tolylamino)ethyl)amino)benzenesulfonamide (5f)

Brown solid. Yield = 38%, silica gel TLC R_f_ 0.50 (EtOAc/Hex 70% *v/v*); ^1^H NMR (400 MHz, DMSO-*d*_6_) *δ* (ppm): 7.54 (d, 2H, *J =* 8.81 Hz), 6.93 (d, 4H, *J =* 6.66 Hz), 6.67 (d, 2H, *J =* 8.81 Hz), 6.54 (d, 2H, *J =* 8.33 Hz), 6.45 (bs, 1H), 5.44 (s, 1H), 3.30 (d, 2H, *J =* 5.34 Hz), 3.23 (d, 2H, *J =* 5.34 Hz), 2.18 (s, 3H); HRMS *m*/*z*: 306.1 [M + H]^+^ in agreement with^34^.

#### 4.6.5. 4-((2-((4-Chlorophenyl)amino)ethyl)amino)benzenesulfonamide (5g)

Black solid. M.p. 178–180 °C. Yield = 53%, silica gel TLC R_f_ 0.23 (MeOH/DCM 3% *v/v*); ^1^H NMR (400 MHz, DMSO-*d*_6_) *δ* (ppm): 7.55 (d, 2H, *J =* 8.67 Hz), 7.13 (d, 2H, *J =* 8.52 Hz), 6.95 (s, 2H), 6.67 (d, 2H, *J =* 8.86 Hz), 6.62 (d, 2H, *J =* 8.86 Hz), 6.46 (t, 1H, *J =* 5.22 Hz), 5.91 (t, 1H, *J =* 5.21 Hz), 3.29 (t, 2H, *J =* 5.65 Hz), 3.25 (t, 2H, *J =* 5.72 Hz); ^13 ^C NMR (100 MHz, DMSO-*d*_6_) *δ* (ppm): 152.4, 148.6, 131.3, 129.7, 128.5, 120.1, 114.5, 111.9, 43.0, 42.7; HRMS *m*/*z*: 326.1 [M + H]^+^.

#### 4.6.6. 4-((2-((4-Iodophenyl)amino)ethyl)amino)benzenesulfonamide (5h)

White solid. Yield = 66%, silica gel TLC R_f_ 0.24 (MeOH/DCM 3% *v/v*); ^1^H NMR (400 MHz, DMSO-*d*_6_) *δ* (ppm): 7.54 (m, 4H), 7.38 (d, 1H, *J =* 8.80 Hz), 6.96 (bs, 2H), 6.68 (m, 4H), 6.49 (d, 1H, *J =* 8.80 Hz), 3.76 (t, 4H, *J =* 6.24 Hz); ^13 ^C NMR (100 MHz, DMSO-*d*_6_) *δ* (ppm): 152.6, 148.8, 131.7, 130.1, 129.5, 120.1, 114.6, 111.7, 43.1, 42.9; HRMS *m*/*z*: 418.0 [M + H]^+^.

#### 4.6.7. 3-((2-(Phenylamino)ethyl)amino)benzenesulfonamide (6l)

Brown solid. M.p. 146–148 °C. Yield = 51%, silica gel TLC R_f_ 0.43 (EtOAc/Hex 50% *v/v*); ^1^H NMR (400 MHz, DMSO-*d*_6_) *δ* (ppm): 7.27 (t, 1H, *J =* 7.80 Hz), 7.21 (s. 1H), 7.12 (t, 2H, *J =* 7.80 Hz), 7.06 (s, 1H), 7.01 (d, 1H, *J =* 7.50 Hz), 6.80 (dd, 1H, *J =* 8.12 Hz, *J =* 6.44 Hz), 6.63 (d, 2H, *J =* 7.79 Hz), 6.57 (t, 1H, *J =* 7.18 Hz), 6.19 (apt, 1H, *J =* 5.19 Hz), 5.66 (apt, 1H, *J =* 5.19 Hz), 3.28 (s, 4H); ^13 ^C NMR (100 MHz, DMSO-*d*_6_) *δ* (ppm): 150.0, 149.7, 145.9, 130.4, 130.0, 116.9, 116.1, 113.6, 113.2, 109.5, 43.1, 42.9; HRMS *m*/*z*: 292.1 [M + H]^+^.

### 4.7. General procedure for compounds 5a,d,i,j and 6k,m–o

To a stirring solution of LiAlH_4_ (192.41 mg, 5.07 mmol, 6 eq.) in dry THF (20 mL) in an ice bath (0 °C), a solution of the suitable amide derivative **3a**,**d**,**i**,**j** or **4k**,**m–o**; (0.85 mmol, 1 eq.) in dry THF (5 ml) was added dropwise under nitrogen atmosphere. Then, the reaction mixture was stirred at 70 °C for 16 h. After cooling in an ice bath at 0 °C, the reaction was quenched with water, and the mixture was extracted with EtOAc three times. The combined organic layers were washed with brine, dried over anhydrous Na_2_SO_4_, filtered, and evaporated under vacuum. The reaction crudes were purified by silica gel flash column chromatography.

#### 4.7.1. 4-((2-(Isopropylamino)ethyl)amino)benzenesulfonamide (5a)

Brown solid. Yield = 59%, silica gel TLC R_f_ 0.10 (MeOH/DCM 15% *v/v*); ^1^H NMR (400 MHz, DMSO-*d*_6_) *δ* (ppm): 7.53 (d, 2H, *J =* 8.26 Hz), 6.94 (s, 2H), 6.65 (d, 2H, *J =* 8.26 Hz), 6.36 (apt, 1H, *J =* 7.40 Hz), 3.15 (m, 2H), 2.72 (m, 2H), 1.66 (m, 1H), 1.01 (d, 6H, *J =* 5.92 Hz); ^13 ^C NMR (100 MHz, DMSO-*d*_6_) *δ* (ppm): 151.9, 130.4, 127.8, 111.1, 48.4, 45.9, 43.4, 23.4; HRMS *m*/*z*: 258.1 [M + H]^+^.

#### 4.7.2. 4-((2-((2-Fluorophenyl)amino)ethyl)amino)benzenesulfonamide (5d)

Brown solid. Yield = 30%, silica gel TLC R_f_ 0.35 (EtOAc/Hex 50% *v/v*); ^1^H NMR (400 MHz, DMSO-*d*_6_) *δ* (ppm): 7.48 (d, 2H, *J =* 8.80 Hz), 6.97 (m, 2H), 6.88 (bs, 2H), 6.73 (m, 1H), 6.62 (d, 2H, *J =* 8.80 Hz), 6.57 (m, 1H), 6.43 (t, 1H, *J =* 5.2 Hz), 5.42 (bs, 1H), 3.26 (m, 4H); HRMS *m*/*z*: 310.1 [M + H]^+^ in agreement with^34^.

#### 4.7.3. 4-((2-((6-Methylpyridin-2-yl)amino)ethyl)amino)benzenesulfonamide (5i)

Brown oil. Yield = 38%, silica gel TLC R_f_ 0.40 (MeOH/DCM 10% *v/v*); ^1^H NMR (400 MHz, DMSO-*d*_6_) *δ* (ppm): 7.55 (d, 2H, *J =* 8.68 Hz), 7.30 (t, 1H, *J =* 7.96 Hz), 6.94 (s, 2H), 6.80 (d, 2H, *J =* 8.90 Hz), 6.57 (apt, 2H, *J =* 7.23 Hz), 6.40 (d, 1H, *J =* 7.23 Hz), 6.30 (d, 1H, *J =* 7.78 Hz), 3.29 (apt, 2H, *J =* 7.10 Hz), 2.35 (s, 3H); ^13 ^C NMR (100 MHz, DMSO-*d*_6_) *δ* (ppm): 148.1, 145.7, 140.8, 130.5, 129.3, 127.3, 119.0, 111.1, 103.5, 49.0, 46.8, 23.9; HRMS *m*/*z*: 307.1 [M + H]^+^.

#### 4.7.4. 4-((2-(Benzylamino)ethyl)amino)benzenesulfonamide (5j)

White solid. Yield = 37%, silica gel TLC R_f_ 0.50 (MeOH/DCM 5% *v/v*); ^1^H NMR (400 MHz, DMSO-*d*_6_) *δ* (ppm): 7.53 (d, 2H, *J =* 8.82 Hz), 7.35 (m, 4H), 7.25 (t, 1H, *J =* 6.74 Hz), 6.94 (s, 2H), 6.65 (d, 2H, *J =* 8.72 Hz), 6.33 (t, 1H, *J =* 5.38 Hz), 3.20 (q, 2H, *J =* 6.04 Hz), 2.73 (t, 2H, *J =* 6.33 Hz); ^13 ^C NMR (100 MHz, DMSO-*d*_6_) *δ* (ppm): 150.8, 140.2, 139.9, 128.5, 127.9, 127.3, 127.0, 111.1, 52.3, 49.4, 48.5; HRMS *m*/*z*: 306.1 [M + H]^+^.

#### 4.7.5. 3-((2-(Butylamino)ethyl)amino)benzenesulfonamide (6k)

Yellow solid. Yield = 32%, silica gel TLC R_f_ 0.50 (EtOAc/Hex 60% *v/v*); ^1^H NMR (400 MHz, DMSO-*d*_6_) *δ* (ppm): 7.25 (t, 1H, *J =* 7.75 Hz), 7.04 (s, 1H), 6.99 (d, 1H, *J =* 7.51 Hz), 6.78 (d, 1H, *J =* 8.00 Hz), 6.05 (apt, 1H, *J =* 5.46 Hz), 3.20 (s, 2H), 3.16 (m, 2H), 2.75 (t, 1H, *J =* 5.96 Hz), 2.12 (s, 2H), 1.14 (m, 2H), 1.13 (m, 2H), 0.90 (t, 3H, *J =* 7.01 Hz);^13^C NMR (100 MHz, DMSO-*d*_6_) *δ* (ppm): 150.1, 145.9, 130.3, 115.9, 113.4, 109.4, 49.7, 48.9, 43.5, 32.5, 20.9, 14.9; HRMS *m*/*z*: 272.1 [M + H]^+^.

#### 4.7.6. 3-((2-((2-Bromophenyl)amino)ethyl)amino)benzenesulfonamide (6m)

Brown solid. M.p. 172–174 °C. Yield = 49%, silica gel TLC R_f_ 0.25 (EtOAc/Hex 40% *v/v*); ^1^H NMR (400 MHz, DMSO-*d*_6_) *δ* (ppm): 7.44 (d, 1H, *J =* 7.94 Hz), 7.27 (m, 2H), 7.19 (s, 2H), 7.07 (s, 1H), 7.01 (d, 2H, *J =* 7.10 Hz), 6.80 (t, 2H, *J =* 9.61 Hz), 6.57 (t, 1H, *J =* 7.40 Hz), 6.28 (t, 1H, *J =* 5.65 Hz), 5.28 (t, 1H, *J =* 5.65 Hz), 3.40 (t, 2H, *J =* 5.70 Hz), 3.31 (t, 2H, *J =* 5.70 Hz); ^13 ^C NMR (100 MHz, DMSO-*d*_6_) *δ* (ppm): 150.3, 145.9, 133.4, 130.5, 129.8, 118.3, 116.0, 113.6, 112.4, 109.5, 42.9, 42.8, 38.3; HRMS *m*/*z*: 370.0 [M + H]^+^.

#### 4.7.7. 3-((2-(p-Tolylamino)ethyl)amino)benzenesulfonamide (6n)

Brown solid. Yield = 42%, silica gel TLC R_f_ 0.50 (EtOAc/Hex 50% *v/v*); ^1^H NMR (400 MHz, DMSO-*d*_6_) *δ* (ppm): 7.27 (t, 1H, *J =* 7.82 Hz), 7.20 (s, 2H), 7.07 (s, 1H), 7.01 (s, 1H, *J =* 7.13 Hz), 6.94 (d, 2H, *J =* 7.82 Hz), 6.80 (d, 1H, *J =* 6.90 Hz), 6.55 (d, 2H, *J =* 8.01 Hz), 6.17 (s, 1H), 5.42 (s, 1H), 3.26 (s, 4H), 2.19 (s, 3H); ^13 ^C NMR (100 MHz, DMSO-*d*_6_) *δ* (ppm): 152.3, 148.4, 131.5, 129.8, 128.5, 120.1, 113.9, 112.5, 43.3, 42.7; HRMS *m*/*z*: 306.1 [M + H]^+^.

#### 4.7.8. 3-((2-((4-Chlorophenyl)amino)ethyl)amino)benzenesulfonamide (6o)

Black solid. M.p. 185–187 °C. Yield = 80%, silica gel TLC R_f_ 0.39 (EtOAc/Hex 50% *v/v*); ^1^H NMR (400 MHz, DMSO-*d*_6_) *δ* (ppm): 7.27 (t, 1H, *J =* 8.02 Hz), 7.20 (bs, 1H), 7.12 (d, 2H, *J =* 8.82 Hz), 7.06 (s, 1H), 7.00 (d, 1H, *J =* 7.62 Hz), 6.91 (s, 1H), 6.79 (d, 1H, *J =* 8.02 Hz), 6.63 (d, 2H, *J =* 8.42 Hz), 6.18 (s, 1H), 5.90 (s, 1H), 3.26 (s, 4H); ^13 ^C NMR (100 MHz, DMSO-*d*_6_) *δ* (ppm): 150.0, 148.6, 145.7, 130.4, 129.7, 120.2, 116.1, 114.5, 113.6, 109.4, 42.9, 31.5; HRMS *m*/*z*: 326.1 [M + H]^+^.

### 4.8. General procedure for compounds 7a–j and 8k–o

To a stirring solution of the appropriate derivative **7a–j** or **8k–o** (0.15 mmol, 1 eq.) in dry THF (10 mL), a solution of triphosgene (22.8 mg, 0.08 mmol, 0.5 eq.) in dry THF (2.5 mL) was added dropwise at r.t. The resulting mixture was stirred at room temperature for 1 h under a nitrogen atmosphere., then dry TEA (21 µL, 0.154 mmol, 1 eq.) was added and the mixture was stirred at room temperature for 1 h under a nitrogen atmosphere. Then, the reaction was quenched with water and extracted with EtOAc three times. The combined organic layers were washed with brine, dried over anhydrous Na_2_SO_4_, filtered, and evaporated under vacuum. The reaction crudes were purified by silica gel flash column chromatography.

#### 4.8.1. 4-(3-Isopropyl-2-oxoimidazolidin-1-yl)benzenesulfonamide (7a)

Brown solid. M.p. 243–245 °C. Yield = 52%, silica gel TLC R_f_ 0.30 (EtOAc/Hex 70% *v/v*); ^1^H NMR (400 MHz, DMSO-*d*_6_) *δ* (ppm): 7.74 (m, 4H), 7.21 (bs, 2H), 3.84 (t, 2H, *J =* 8.44 Hz), 3.46 (t, 2H, *J =* 8.12 Hz); ^13 ^C NMR (100 MHz, DMSO-*d*_6_) *δ* (ppm): 156.4, 144.0, 136.8, 126.9, 116.4, 43.7, 42.4, 36.3, 19.7; HRMS *m*/*z*: 284.1 [M + H]^+^.

#### 4.8.2. 4-(3-Butyl-2-oxoimidazolidin-1-yl)benzenesulfonamide (7b)

Brown solid. M.p. 274–276 °C. Yield = 38%, silica gel TLC R_f_ 0.1 (MeOH/DCM 10% *v/v*); ^1^H NMR (400 MHz, DMSO-*d*_6_) *δ* (ppm): 7.81 (m, 4H), 7.27 (s, 2H), 1.45 (m, 2H), 1.36 (m, 2H), 1.13 (t, 2H, *J =* 7.16 Hz), 0.92 (t, 3H, *J =* 7.40 Hz); ^13 ^C NMR (100 MHz, DMSO-*d*_6_) *δ* (ppm): 159.1, 146.7, 136.5, 129.4, 121.9, 49.5, 41.8, 29.5, 26.4, 19.7, 13.8; HRMS *m*/*z*: 298.1 [M + H]^+^.

#### 4.8.3. 4-(2-Oxo-3-phenylimidazolidin-1-yl)benzenesulfonamide (7c)

White solid. Yield = 63%, silica gel TLC R_f_ 0.31 (MeOH/DCM 5% *v/v*); ^1^H NMR (400 MHz, DMSO-*d*_6_) *δ* (ppm): 7.84 (s, 4H), 7.68 (s, 2H, *J =* 8.24 Hz), 7.43 (t, 2H, *J =* 7.76 Hz), 7.30 (s, 2H), 7.14 (t, 1H, *J =* 7.27 Hz), 4.06 (s, 4H); ^13 ^C NMR (100 MHz, DMSO-*d*_6_) *δ* (ppm): 155.3, 144.1, 140.9, 138.5, 129.8, 127.6, 123.9, 119.1, 118.1, 42.5; HRMS *m*/*z*: 318.1 [M + H]^+^.

#### 4.8.4. 4-(3-(2-Fluorophenyl)-2-oxoimidazolidin-1-yl)benzenesulfonamide (7d)

Brown solid. M.p. 221–223 °C. Yield = 73%, silica gel TLC R_f_ 0.45 (EtOAc/DCM 70% *v/v*); ^1^H NMR (400 MHz, DMSO-*d*_6_) *δ* (ppm): 7.81 (m, 4H), 7.58 (m, 1H), 7.31 (m, 2H), 7.21 (m, 2H), 4.05 (m, 2H), 3.95 (m, 2H); HRMS *m*/*z*: 336.1 [M + H]^+^ in agreement with^34^.

#### 4.8.5. 4-(3-(2-Bromophenyl)-2-oxoimidazolidin-1-yl)benzenesulfonamide (7e)

Brown solid. M.p. 241–243 °C. Yield = 95%, silica gel TLC R_f_ 0.30 (MeOH/DCM 3% *v/v*); ^1^H NMR (400 MHz, DMSO-*d*_6_) *δ* (ppm): 7.83 (m, 5H), 7.60 (dd, 1H, *J =* 7.89 Hz, *J =* 6.44 Hz), 7.53 (t, 1H, *J =* 7.89 Hz), 7.37 (m, 1H), 7.29 (s, 2H), 4.12 (t, 2H, *J =* 8.42 Hz), 3.94 (t, 2H, *J =* 8.42 Hz); ^13 ^C NMR (100 MHz, DMSO-*d*_6_) *δ* (ppm): 155.5, 143.5, 138.4, 197.7, 133.7, 130.8, 130.1, 129.3, 127.0, 123.1, 117.0, 44.2, 42.9; HRMS *m*/*z*: 396.0 [M + H]^+^.

#### 4.8.6. 4-(2-Oxo-3-(p-tolyl)imidazolidin-1-yl)benzenesulfonamide (7f)

Brown solid. M.p. 279–281 °C. Yield = 27%, silica gel TLC R_f_ 0.32 (MeOH/DCM 5% *v/v*); ^1^H NMR (400 MHz, DMSO-*d*_6_) *δ* (ppm): 7.84 (s, 4H), 7.56 (d, 2H, *J =* 8.56 Hz), 7.29 (s, 2H), 7.23 (d, 2H, *J =* 8.56 Hz), 4.03 (s, 4H), 2.33 (s, 3H); HRMS *m*/*z*: 332.1 [M + H]^+^ in agreement with^34^.

#### 4.8.7. 4-(3-(4-Chlorophenyl)-2-oxoimidazolidin-1-yl)benzenesulfonamide (7g)

Black solid. M.p. 267–269 °C. Yield = 35%, silica gel TLC R_f_ 0.55 (MeOH/DCM 3% *v/v*); ^1^H NMR (400 MHz, DMSO-*d*_6_) *δ* (ppm): 7.84 (s, 4H), 7.72 (d, 2H, *J =* 9.12 Hz), 7.48 (d, 2H, *J =* 9.12 Hz), 7.30 (s, 2H), 4.05 (s, 4H); ^13 ^C NMR (100 MHz, DMSO-*d*_6_) *δ* (ppm): 155.1, 143.8, 139.8, 138.6, 129.6, 127.6, 125.9, 120.5, 118.3, 42.4, 31.5; HRMS *m*/*z*: 352.0 [M + H]^+^.

#### 4.8.8. 4-(3-(4-Iodophenyl)-2-oxoimidazolidin-1-yl)benzenesulfonamide (7h)

Brown solid. M.p. 250–252 °C. Yield = 45%, silica gel TLC R_f_ 0.47 (EtOAc/Hex 60% *v/v*); ^1^H NMR (400 MHz, DMSO-*d*_6_) *δ* (ppm): 7.55 (d, 4H, *J =* 8.68 Hz), 6.97 (bs, 2H), 6.71 (d, 4H, *J =* 8.67 Hz), 3.75 (t, 2H, *J =* 6.19 Hz), 3.50 (t, 2H, *J =* 6.19 Hz); ^13 ^C NMR (100 MHz, DMSO-*d*_6_) *δ* (ppm): 154.5, 143.2, 139.2, 137.9, 129.0, 127.0, 119.9, 117.5, 114.4, 41.8, 30.9; HRMS *m*/*z*: 444.0 [M + H]^+^.

#### 4.8.9. 4-(3-(6-Methylpyridin-2-yl)-2-oxoimidazolidin-1-yl)benzenesulfonamide (7i)

Yellow solid. Yield = 34%, silica gel TLC R_f_ 0.40 (EtOAc/Hex 70% *v/v*); ^1^H NMR (400 MHz, DMSO-*d*_6_) *δ* (ppm): 8.05 (d, 2H, *J =* 8.27 Hz), 7.84 (s, 4H), 7.71 (t, 1H, *J =* 7.85 Hz), 7.28 (s, 2H), 4.14 (t, 2H, *J =* 8.63 Hz), 4.02 (t, 2H, *J =* 8.40 Hz); ^13 ^C NMR (100 MHz, DMSO-*d*_6_) *δ* (ppm): 155.7, 154.6, 152.3, 146.7, 138.5, 136.5, 129.4, 121.9, 119.0, 26.0, 23.9; HRMS *m*/*z*: 333.1 [M + H]^+^.

#### 4.8.10. 4-(3-Benzyl-2-oxoimidazolidin-1-yl)benzenesulfonamide (7j)

White solid. M.p. 244–246 °C. Yield = 65%, silica gel TLC R_f_ 0.38 (EtOAc/Hex 70% *v/v*); ^1^H NMR (400 MHz, DMSO-*d*_6_) *δ* (ppm): 7.78 (s, 4H), 7.39 (t, 2H, *J =* 7.31 Hz), 7.33 (d, 3H, *J =* 7.52 Hz), 7.22 (s, 2H), 4.44 (s, 2H), 3.88 (t, 2H, *J =* 8.42 Hz), 3.42 (t, 2H, *J =* 7.77 Hz);^13^C NMR (100 MHz, DMSO-*d*_6_) *δ* (ppm): 157.8, 144.5, 138.0, 137.7, 129.7, 128.9, 128.4, 127.6, 117.2, 48.1, 42.9, 41.8; HRMS *m*/*z*: 332.1 [M + H]^+^.

#### 4.8.11. 3-(3-Butyl-2-oxoimidazolidin-1-yl)benzenesulfonamide (8k)

Brown solid. M.p. 177–179 °C. Yield = 37%, silica gel TLC R_f_ 0.40 (EtOAc/Hex 60% *v/v*); ^1^H NMR (400 MHz, DMSO-*d*_6_) *δ* (ppm): 8.18 (s, 1H), 7.71 (d, 1H, *J =* 8.26 Hz), 7.51 (t, 1H, *J =* 8.26 Hz), 7.46 (d, 1H, *J =* 7.80 Hz), 7.36 (s, 2H), 3.86 (t, 2H, *J =* 8.49 Hz), 3.52 (t, 2H, *J =* 8.20 Hz), 3.24 (t, 2H, *J =* 7.07 Hz), 1.53 (t, 2H, *J =* 7.23 Hz), 1.34 (m, 2H), 0.95 (t, 3H, *J =* 7.43 Hz); ^13 ^C NMR (100 MHz, DMSO-*d*_6_) *δ* (ppm): 157.9, 145.6, 142.2, 130.3, 120.4, 119.4, 114.7, 43.8, 43.0, 41.9, 29.9, 20.5. 14.7; HRMS *m*/*z*: 298.1 [M + H]^+^.

#### 4.8.12. 3-(2-Oxo-3-phenylimidazolidin-1-yl)benzenesulfonamide (8l)

Brown solid. M.p. 215–217 °C. Yield = 51%, silica gel TLC R_f_ 0.31 (MeOH/DCM 5% *v/v*); ^1^H NMR (400 MHz, DMSO-*d*_6_) *δ* (ppm): 8.26 (s, 1H), 7.79 (d, 1H, *J =* 7.79 Hz), 7.68 (d, 2H, *J =* 8.08 Hz), 7.58 (m, 2H), 7.42 (m, 4H), 7.13 (t, 1H, *J =* 7.27 Hz), 4.05 (s, 4H); ^13 ^C NMR (100 MHz, DMSO-*d*_6_) *δ* (ppm): 155.3, 145.7, 141.6, 140.9, 130.4, 129.8, 123.8, 121.4, 120.3, 118.9, 115.7, 42.5; HRMS *m*/*z*: 318.1 [M + H]^+^.

#### 4.8.13. 3-(3-(2-Bromophenyl)-2-oxoimidazolidin-1-yl)benzenesulfonamide (8m)

Brown solid. Yield = 68%, silica gel TLC R_f_ 0.57 (EtOAc/Hex 70% *v/v*); ^1^H NMR (400 MHz, DMSO-*d*_6_) *δ* (ppm): 8.22 (s, 1H), 7.77 (apt, 2H, *J =* 7.39 Hz), 7.53 (m, 4H), 7.38 (bs, 3H), 4.08 (apt, 2H, *J =* 5.54 Hz), 3.93 (apt, 2H, *J =* 5.54 Hz); ^13 ^C NMR (100 MHz, DMSO-*d*_6_) *δ* (ppm): 156.3, 145.7, 141.7, 139.0, 134.3, 131.3, 130.7, 130.5, 129.9, 123.7, 121.0, 120.2, 115.3, 44.9, 43.7; HRMS *m*/*z*: 395.9 [M + H]^+^.

#### 4.8.14. 3-(2-Oxo-3-(p-tolyl)imidazolidin-1-yl)benzenesulfonamide (8n)

Brown solid. M.p. 183–185 °C. Yield = 37%, silica gel TLC R_f_ 0.48 (EtOAc/Hex 50% *v/v*); ^1^H NMR (400 MHz, DMSO-*d*_6_) *δ* (ppm): 8.26 (s, 1H), 7.78 (d, 1H, *J =* 9.07 Hz), 7.57 (m, 4H), 7.42 (s, 2H), 7.22 (d, 2H, *J =* 8.00 Hz), 4.02 (s, 4H), 2.23 (s, 3H); ^13 ^C NMR (100 MHz, DMSO-*d*_6_) *δ* (ppm): 154.7, 145.1, 141.0, 137.9, 132.2, 129.8, 129.6, 120.6, 119.6, 118.4, 114.9, 41.9, 20.8; HRMS *m*/*z*: 332.1 [M + H]^+^.

#### 4.8.15. 3-(3-(4-Chlorophenyl)-2-oxoimidazolidin-1-yl)benzenesulfonamide (8o)

Black solid. M.p. 185–187 °C. Yield = 60%, silica gel TLC R_f_ 0.40 (EtOAc/Hex 70% *v/v*); ^1^H NMR (400 MHz, DMSO-*d*_6_) *δ* (ppm): 8.26 (s, 1H), 7.79 (d, 1H, *J =* 7.85 Hz), 7.72 (dd, 2H, *J =* 8.98 Hz, *J =* 6.54 Hz), 7.58 (m, 2H), 7.47 (dd, 2H, *J =* 9.07 Hz, *J =* 6.46 Hz), 7.42 (s, 2H), 4.05 (s, 4H); ^13 ^C NMR (100 MHz, DMSO-*d*_6_) *δ* (ppm): 154.6, 145.1, 140.8, 139.3, 129.9, 129.0, 126.9, 120.8, 119.9, 119.8, 115.1, 41.9; HRMS *m*/*z*: 352.0 [M + H]^+^.

### 4.9. *In vitro* carbonic anhydrase inhibition assay

The CA-catalyzed CO_2_ hydration activity was performed on an Applied Photophysics stopped-flow instrument using phenol red, at a concentration of 0.2 mM, as a pH indicator with 20 mM HEPES (pH 7.5) as the buffer, 20 mM Na_2_SO_4_, and following the initial rates of the CA-catalyzed CO_2_ hydration reaction for a period of 10–100 s and working at the maximum absorbance of 557 nm. The CO_2_ concentrations ranged from 1.7 to 17 mM. For each inhibitor, six traces of the initial 5−10% of the reaction have been used to determine the initial velocity. The uncatalyzed reaction rates were determined in the same manner and subtracted from the total observed rates. Stock solutions of inhibitor (0.1 mM) were prepared in distilled water, and dilutions up to 0.01 nM were prepared. Solutions containing inhibitor and enzyme were preincubated for 15 min at room temperature before assay to allow the formation of the E−I complex. The inhibition constants were obtained as non-linear least-squares protocols using PRISM 3 and are the mean from at least three different measurements. All CAs were recombinant ones and were obtained in house^42^^,^^43^.

### 4.10. Cell culture and reagents

Human U87MG (ATCC HTB-14), U251 (Merck U-251 MG), T98G (ATCC CRL-1690) cells and human breast cancer MDA-MB-468 cells (ATCC HTB-132) and MCF-7 (ATCC HTB-2) were grown in DMEM supplemented with 10% FCS. Human pancreatic cancer CF-PAC-1 (ATCC CRL-1918) and PANC-1 (ATCC CRL-1469) cells were grown in RPMI 1640 supplemented with 10% FCS. Cells were kept at the low passage, returning to original frozen stocks every 3–4 months. Hypoxic culture conditions were realised in the presence of 1% O_2_ and 5% CO_2_.

### 4.11. Quantitative PCR

Cells were cultured at 37 °C with 1% O_2_ and 5% CO_2_ for 24 h. Total RNA was isolated using TRIzol Reagent (Invitrogen) according to the manufacturer's instructions. Two μg of total RNA were retro-transcribed with MMLV reverse transcriptase (Invitrogen) using random primers. cDNA was analysed by quantitative real-time polymerase chain reaction (qPCR) analysis. β-Actin was used as a housekeeping gene for normalisation. Primers used: Hs-CAIX forward: 5′-CTTTGAATGGGCGAGTGATT-3′, reverse: 5′-TTCTGTGCTGCCTTCTCATCT-3′; Hs-B-actin forward: 5′-CACACAGGGGAGGTGATAGC-3′, reverse: 5′-GACCAAAAGCCTTCATACATCTCA-3′.

### 4.12. Cell proliferation assay

The different cellular lines were seeded in 48-well plates and treated in 1% FBS with increasing concentrations of compounds **7a**, **7d**, and **8m** or **SLC-0111**. After 72 h of incubation at 37 °C with 1% O_2_, 5% CO_2_, and 94% N_2_, cells were trypsinized and cell counting was performed with the MACSQuant^®^ Analyser (Miltenyi Biotec)^44^.

## Author contributions

The manuscript was written with the contributions of all authors. All authors have approved the final version of the manuscript.

## Supplementary Material

Supplemental Material
