# Supplementary material for: Benzenesulfonamides with different rigidity-conferring linkers as carbonic anhydrase inhibitors: an insight into the antiproliferative effect on glioblastoma, pancreatic, and breast cancer cells
Source: J Enzyme Inhib Med Chem. 2022 Jun 29;37(1):1857–69. doi: 10.1080/14756366.2022.2091557 (PMC9246135; doi:10.1080/14756366.2022.2091557)
Supplement: Supplemental Material [file IENZ_A_2091557_SM6456.pdf]

Supporting information

Benzenesulfonamides with different rigidity-conferring linkers as carbonic anhydrase inhibitors: An insight into the antiproliferative effect on glioblastoma, pancreatic, and breast cancer cells

Francesco Liguori[a,b], Simone Carradori[a,*], Fabrizio Carta[b], Serena Filiberti[c], Sara Rezzola[c], Roberto Ronca[c,*],Claudiu T. Supuran[b]

[a]Department of Pharmacy, "G. d'Annunzio" University of Chieti-Pescara, via dei Vestini 31, 66100 Chieti, Italy.

[b]Neurofarba Department, University of Florence, 50019 Sesto Fiorentino (Florence), Italy.

[c]Department of Molecular and Translational Medicine, University of Brescia, Viale Europa 11, 25123 Brescia, Italy.

[*]Simone Carradori, simone.carradori@unich.it; Roberto Ronca, roberto.ronca@unibs.it

2-(butylamino)-*N*-(4-sulfamoylphenyl)acetamide (3b)

^{1}H NMR (400 MHz, DMSO-d_6)

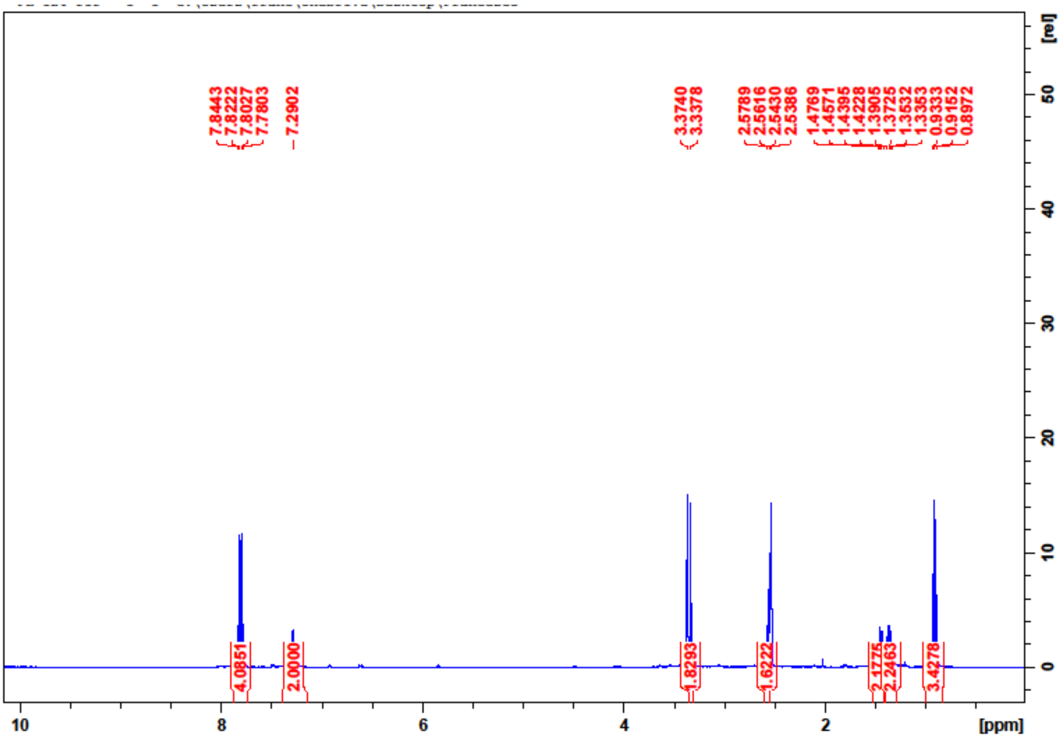

2-(phenylamino)-*N*-(4-sulfamoylphenyl)acetamide (3c)

^{1}H NMR (400 MHz, DMSO-d_6)

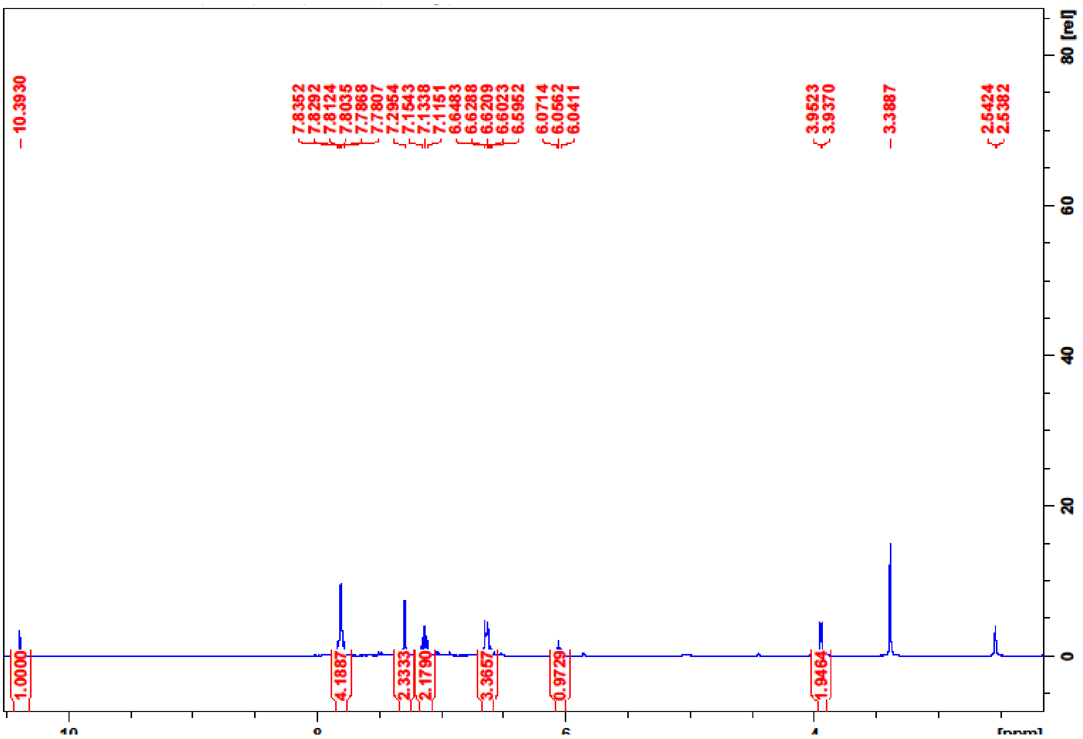

2-((2-fluorophenyl)amino)-*N*-(4-sulfamoylphenyl)acetamide (3d)

^{1}H NMR (400 MHz, DMSO-d_6)

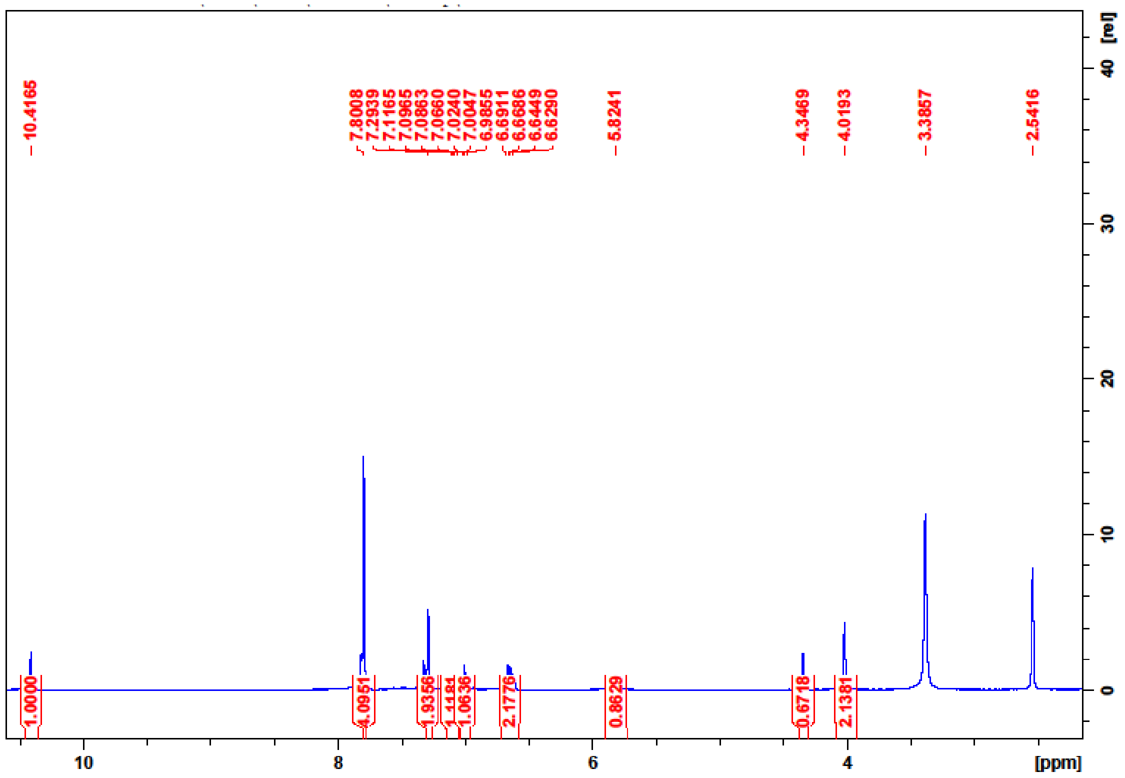

2-((4-chlorophenyl)amino)-*N*-(3-sulfamoylphenyl)acetamide (4o)

^{1}H NMR (400 MHz, DMSO-d_6)

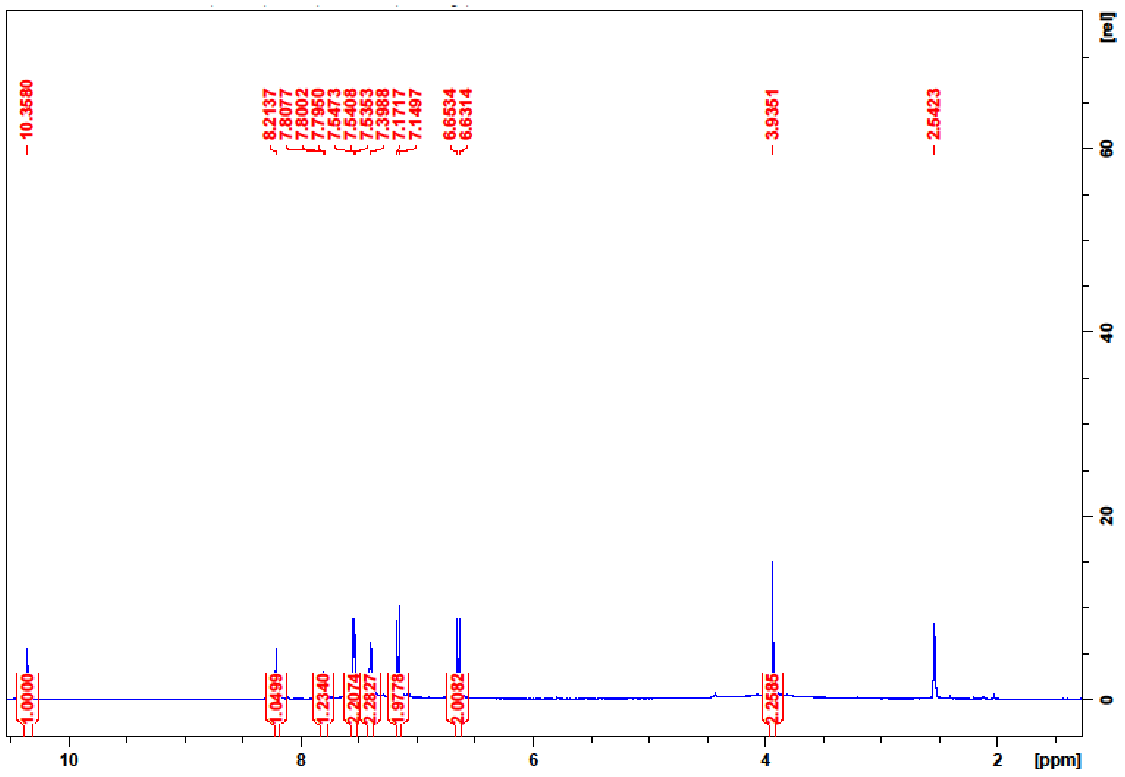

4-((2-(butylamino)ethyl)amino)benzenesulfonamide (5b)

^{1}H NMR (400 MHz, DMSO-d_6)

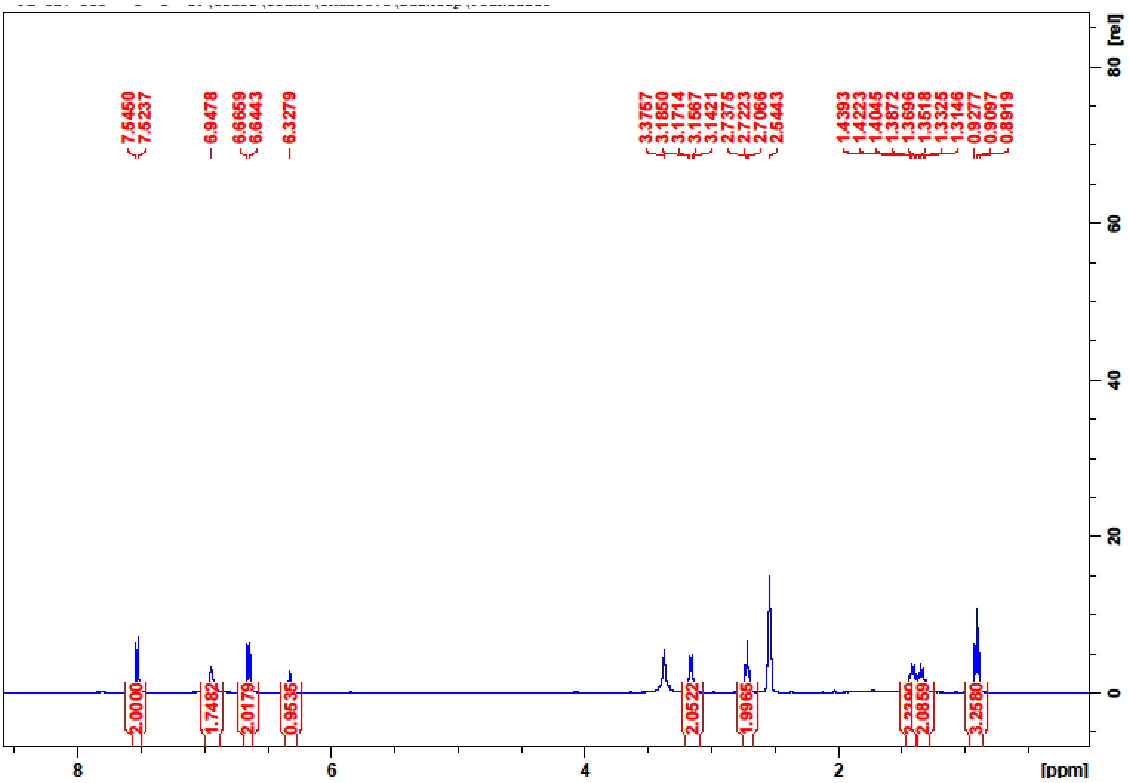

4-(3-isopropyl-2-oxoimidazolidin-1-yl)benzenesulfonamide (7a)

^{1}H NMR (400 MHz, DMSO-d_6)

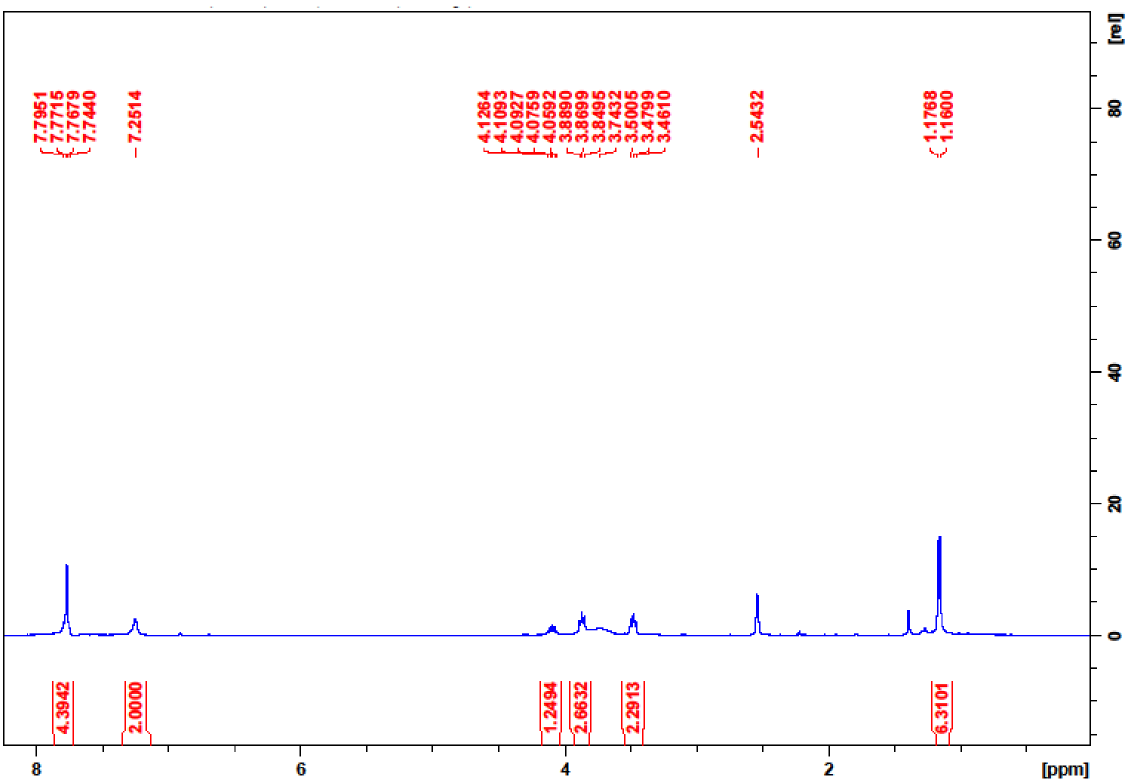

4-(3-(2-fluorophenyl)-2-oxoimidazolidin-1-yl)benzenesulfonamide (7d)

^{1}H NMR (400 MHz, DMSO-d_6)

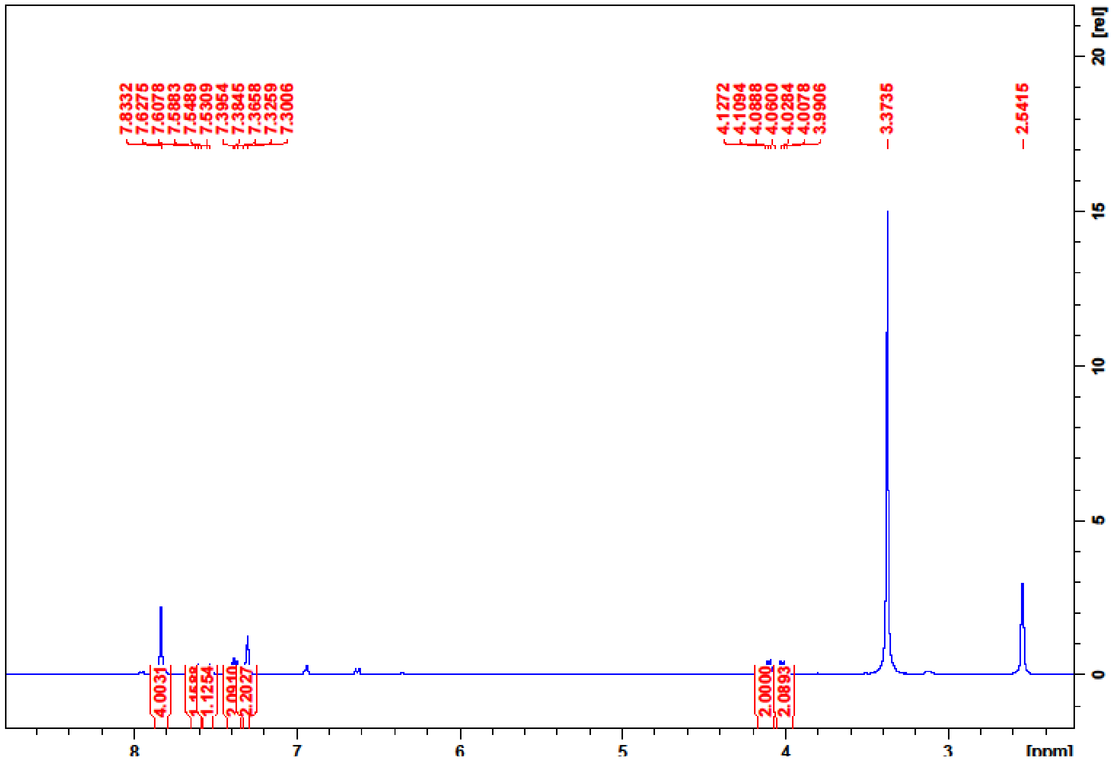

4-(3-(2-bromophenyl)-2-oxoimidazolidin-1-yl)benzenesulfonamide (7e)

^{1}H NMR (400 MHz, DMSO-d_6)

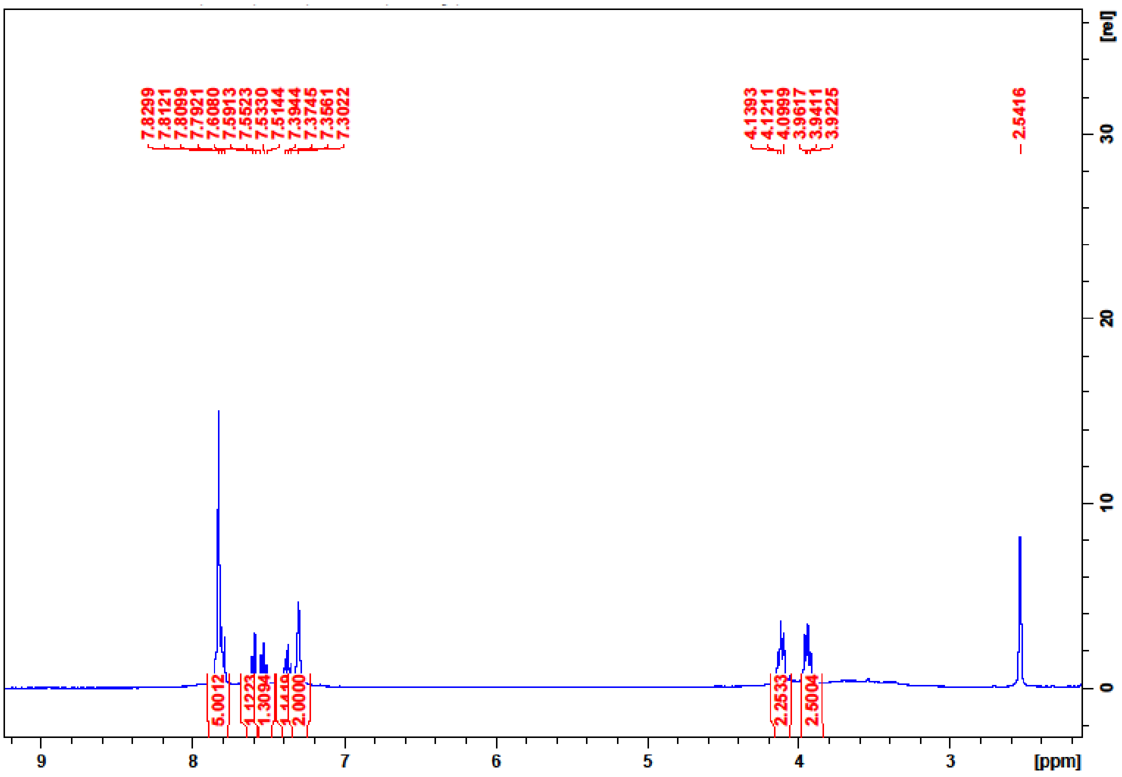

4-(2-oxo-3-(p-tolyl)imidazolidin-1-yl)benzenesulfonamide (7f)

^{1}H NMR (400 MHz, DMSO-d_6)

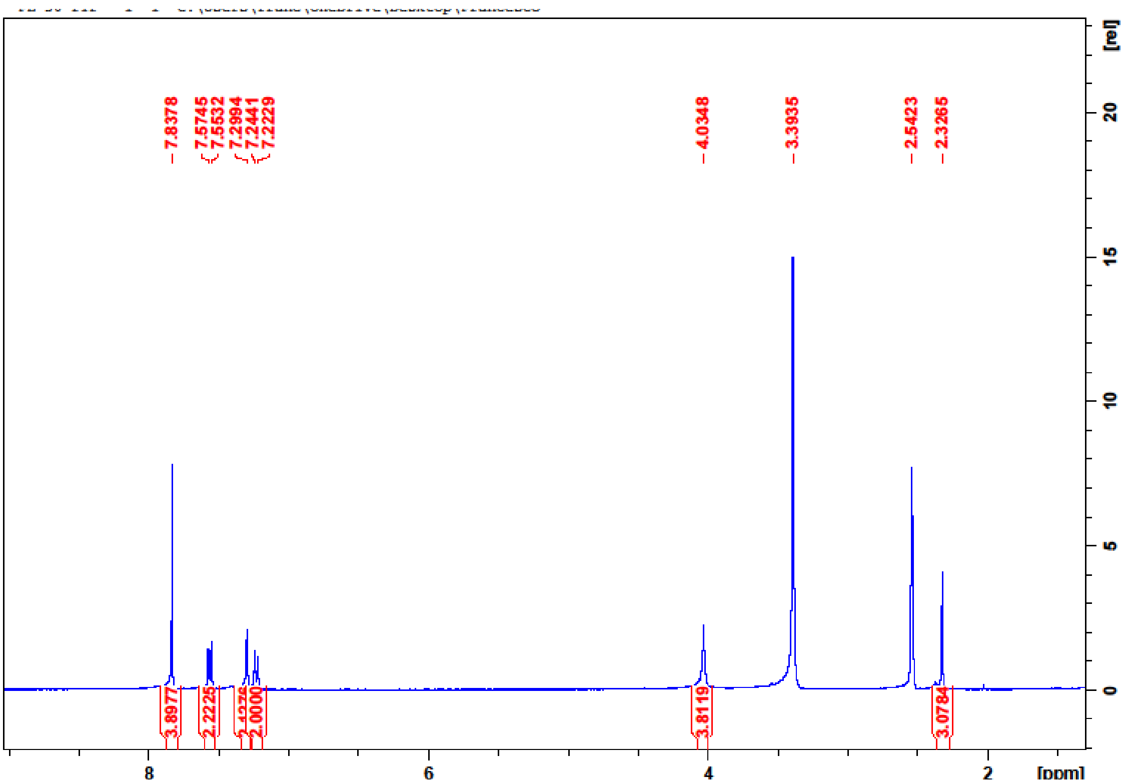

4-(3-(6-methylpyridin-2-yl)-2-oxoimidazolidin-1-yl)benzenesulfonamide (7i)

^{1}H NMR (400 MHz, DMSO-d_6)

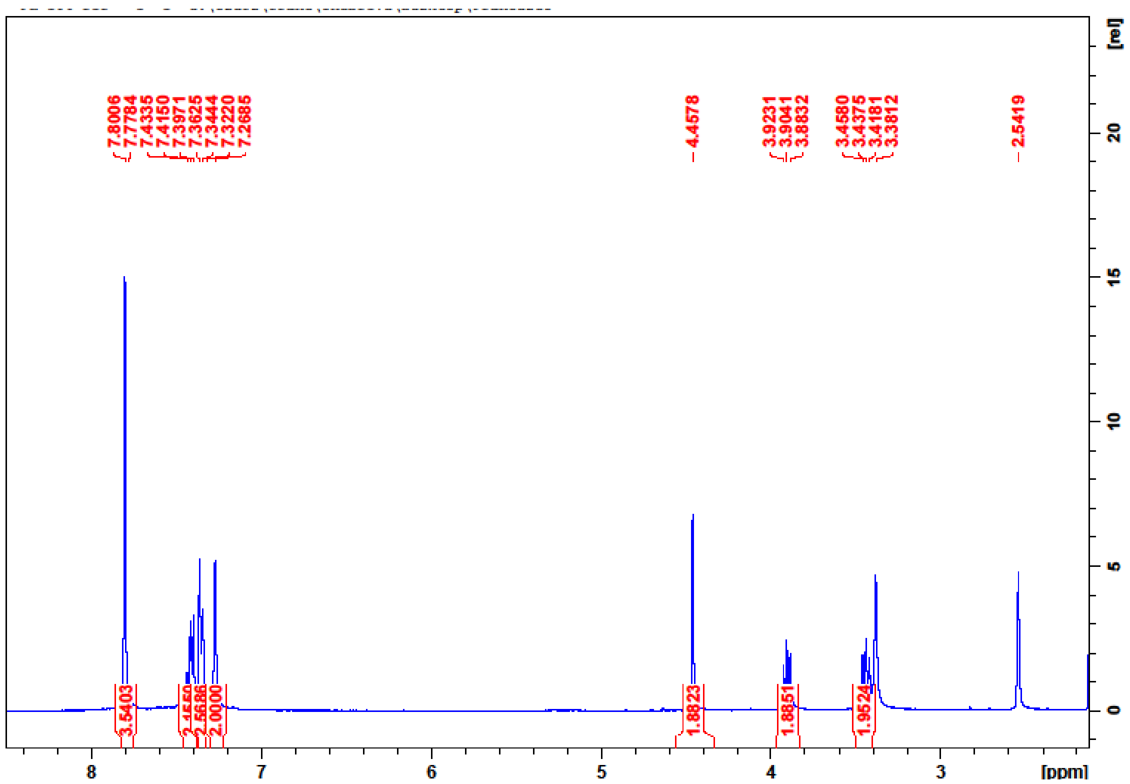

3-(3-(2-bromophenyl)-2-oxoimidazolidin-1-yl)benzenesulfonamide (8m)

^{1}H NMR (400 MHz, DMSO-d_6)

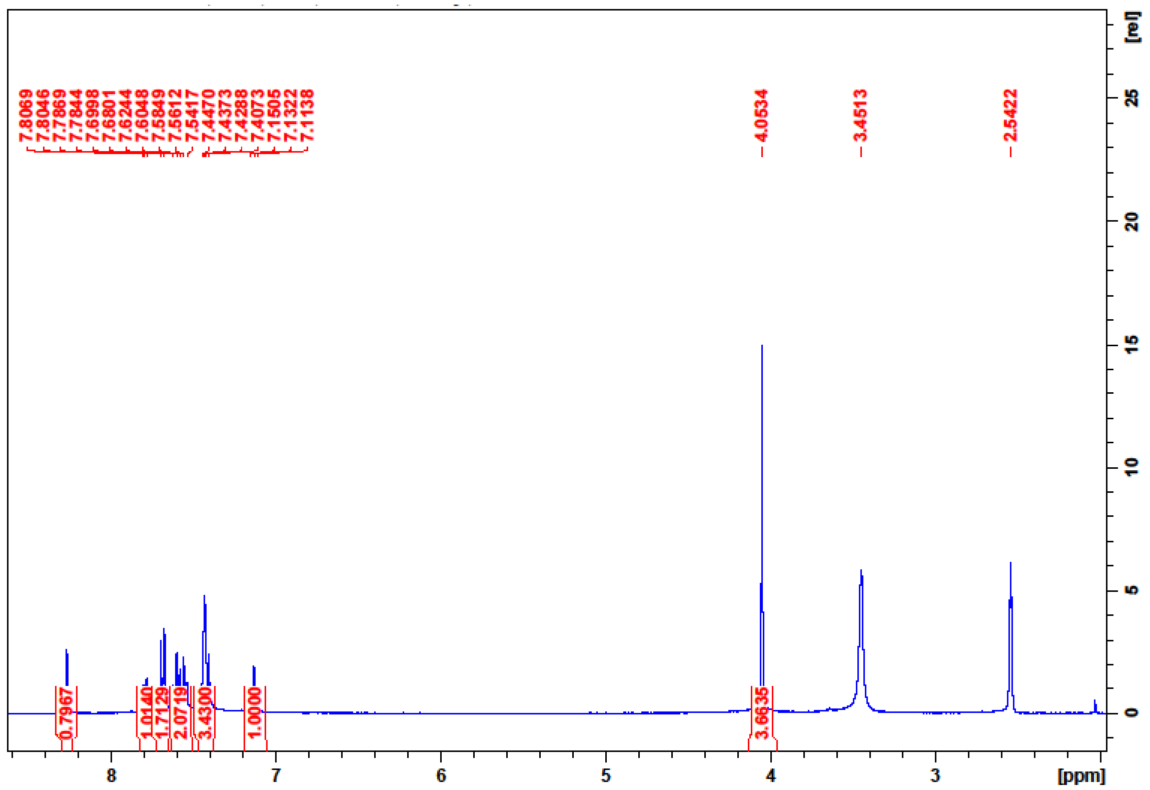

3-(2-oxo-3-(p-tolyl)imidazolidin-1-yl)benzenesulfonamide (8n)

^{1}H NMR (400 MHz, DMSO-d_6)

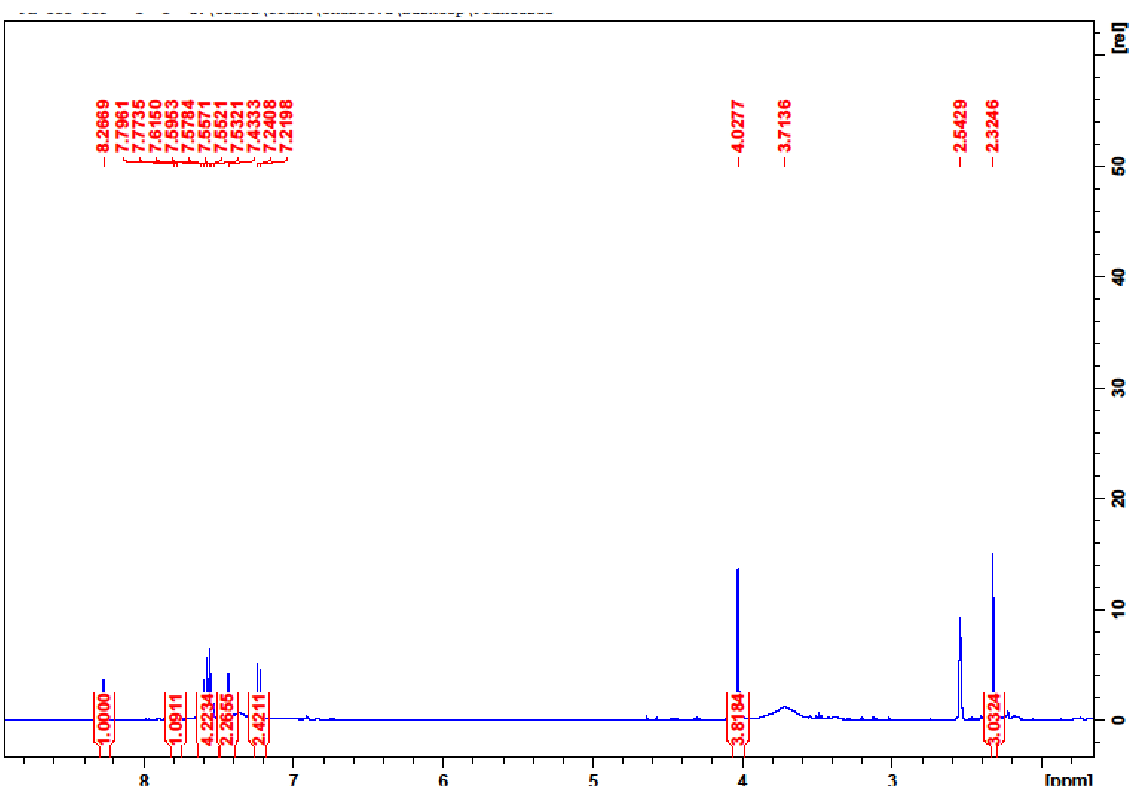

8.2669
7.7961
7.7735
7.6150
7.5953
7.5784
7.5571
7.5521
7.5321
7.4333
7.2408
7.2198
4.0277
3.7136
2.5429
2.3246
1.0000
1.0911
4.2234
2.2655
2.4211
3.8184
3.0324
[rel]
[ppm]
